# Physiological impact and comparison of mutant screening methods in *piwil2* KO founder Nile tilapia produced by CRISPR/Cas9 system

**DOI:** 10.1038/s41598-020-69421-0

**Published:** 2020-07-28

**Authors:** Ye Hwa Jin, Baoshan Liao, Herve Migaud, Andrew Davie

**Affiliations:** 1 The Institute of Aquaculture, Faculty of Natural Sciences, University of Stirling, Stirling, UK; 2 Present Address: The Roslin Institute and Royal (Dick) School of Veterinary Studies, University of Edinburgh, Edinburgh, UK; 3 Present Address: Department of Applied Biology and Chemical Technology, The Hong Kong Polytechnic University, Hung Hom, Hong Kong

**Keywords:** Ichthyology, CRISPR-Cas9 genome editing, Reproductive biology

## Abstract

The application of genome engineering techniques to understand the mechanisms that regulate germ cell development opens promising new avenues to develop methods to control sexual maturation and mitigate associated detrimental effects in fish. In this study, the functional role of *piwil2* in primordial germ cells (PGCs) was investigated in Nile tilapia using CRISPR/Cas9 and the resultant genotypes were further explored. *piwil2* is a gonad-specific and maternally deposited gene in Nile tilapia eggs which is known to play a role in repression of transposon elements and is therefore thought to be important for maintaining germline cell fate. A functional domain of *piwil2,* PIWI domain, was targeted by injecting Cas9 mRNA and sgRNAs into Nile tilapia embryos at 1 cell stage. Results showed 54% of injected mutant larvae had no or less putative PGCs compared to control fish, suggesting an essential role of *piwil2* in survival of PGCs. The genotypic features of the different phenotypic groups were explored by next generation sequencing (NGS) and other mutant screening methods including T7 endonuclease 1 (T7E1), CRISPR/Cas-derived RNA-guided engineered nuclease (RGEN), high resolution melt curve analysis (HRMA) and fragment analysis. Linking phenotypes to genotypes in F0 was hindered by the complex mosacism and wide indel spectrum revealed by NGS and fragment analysis. This study strongly suggests the functional importance of *piwil2* in PGCs survival. Further studies should focus on reducing mosaicism when using CRISPR/Cas9 system to facilitate direct functional analysis in F0.

## Introduction

Nile tilapia (*Oreochromis niloticus*) is one of the fastest growing farmed finfish species with > 120% increase in production volume over the last decade, such that global production has exceeded 4.1 million tonnes, worth USD 7.6 billion in 2017^1^ making it the second largest (by volume) farmed finfish globally. While Nile tilapia is native to Northern Africa and Israel, it is now farmed widely out with its native range in many countries, contributing significantly to global food security particularly for poor rural communities^2^. A significant hurdle that limited production potential of the species is precocious maturation where individuals direct energy towards sexual maturation to the detriment of somatic growth, which also results in the overproduction of unmarketable fry^3^. This challenge has largely been overcome with the production of all male stocks which result in a more efficient production of tilapia with increased harvest weight^4^. While this has had a significant beneficial impact on production of the species, contributing to its rapid expansion globally, the farming of reproductively competent animals has also resulted in widespread environmental impacts. Nile tilapia is considered to be an established invasive species in Asia^5^ as well as Australia and North and South America, with reported impacts on native species and ecosystems^6^. Therefore, there remains a need to develop methodologies to induce sterility and reduce production losses associated with precocious maturation while also mitigating the potential environmental impacts of farming across the globe.

There has been a recent rise in the application of gene editing approaches using CRISPR/Cas9 to induce KO-mutations associated with a range of phenotypes in tilapia^7–11^ including work directed towards the disruption of primordial germ cells (PGCs) to both better understand the regulation of germline cells and explore the feasibility of creating sterile fish^12,13^. We have previously screened 11 candidate genes and identified 5 putative targets (*nanos3, piwil1, piwil2, dnd1* and *vasa*) for gene KO to induce sterility in Nile tilapia^14^. To date only *nanos2* and *nanos3* have been investigated in this context with CRISPR/Cas9 KO larvae showing an apparent lack of PGCs at the hatching stage^15^. In the present study, we have selected the *piwi-like 2* (*piwil2*) gene based on its gonad-specificity and maternal deposition in Nile tilapia^14^ in addtion to its association with early stages of gametogenesis in mice^16^. The *piwi* gene family has two distinct domains, the PAZ domain, an RNA binding motif, and the PIWI domain, a similar structure to the RNase H catalytic domain^17^. The PIWI domain is known to act as a catalytic engine in RNA-induced silencing complexes (RISC) for RNA interference^18^ and for that reason was targeted in this study through the injection of CRISPR/Cas9 constructs into the embryonic cell of Nile tilapia zygote at the 1-cell stage. The subsequent physiological impact of *piwil2* KO on PGCs was evaluated histologically in hatched larvae.

One of the challenges of applying CRISPR/Cas9 methodologies in poikilothermic species like fish is that temperature conditions in vivo will be suboptimal for *Streptococcus pyogenes* Cas9 (SpCas9) activity which is optimal at 37 °C^19^. Although editing activity with the CRISPR/Cas9 has been reported in tropical species like zebrafish, medaka and Nile tilapia at 26–28 °C^13,15,20^, the apparent diversity in resultant individual genotypes requires careful analysis and interpretation. To date, CRISPR/Cas9-mediated sterility studies have lacked a comprehensive screening and understanding of genotypes generated in F0 animals due to biased mutant screening methods, lack of standardisation and/or methodological details. The lack of understanding of the resultant mutations including the level of mosaicism and the indel spectrum hinders the direct functional analysis in the injected animals. The most frequently used screening methods in gonad-related gene functional studies have been restriction enzyme digestion (RED) and Sanger sequencing of a limited number of cloned sequences^8–13,15,21–23^, with a few studies adopting high resolution melt curve analysis (HRMA) or SURVEYOR techniques^24,25^. Such approaches have a number of potential limitations in their accuracy to describe KO effects on target genotypes, which are further confounded by the pooling of samples which ultimately provides a biased interpretation of the efficacy of CRISPR/Cas9 gene editing in this field. Therefore, this study was also designed to compare and validate the different mutant screening methods in individual F0 fish using targeted next generation sequencing (NGS) as a reference compared to T7 endonuclease I (T7EI), CRISPR/Cas-derived RNA-guided engineered nuclease (RGEN) assay, HRMA and fragment analysis to validate approriate and informative assessment methods.

Here we have applied an iterative approach to optimise the CRISPR/Cas9 KO of *piwil2* in Nile tilapia. The phenotypic impact was analysed histologically and the resultant genotypes were described by NGS, and the accuracy of the mutant screening was compared using a range of methods reported in the literature. A wide indel diversity and high mosaicism was reported in *piwil2* KO F0 animals produced by CRISPR/Cas9 and non-homologous end joining (NHEJ) and microhomology-mediated end joining (MMEJ) DNA repairs were revealed by deep sequencing. Overall, the result reported in the present study provides new insights into the functional importance of *piwil2* in PGC survival as well as the indel diversity and the level of mosaicism produced by CRISPR/Cas9 that are important for the selection of suitable mutant screening methods in future gene editing studies.

## Results

### Mutation frequency of piwil2 sgRNA1 and sgRNA2 at three different concentrations

**Figure 1:**
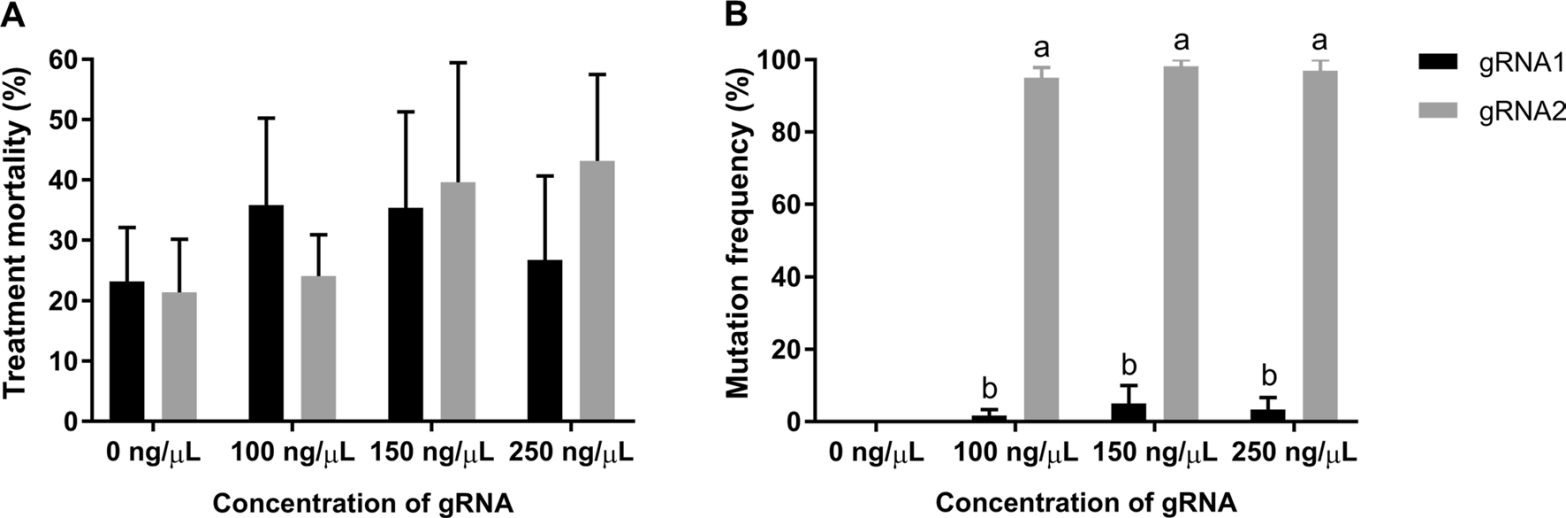
Treatment mortality and mutation frequency in embryos injected with different concentrations of sgRNAs. (**A**) Treatment mortality and (**B**) mutation frequency induced by different concentrations of *piwil2* sgRNA1 or sgRNA2 together with 500 ng/µL of Cas9 mRNAs. Data were collected from three independent egg batches and the treatment mortality was recorded at 3 dpf. Putative mutants were screened by qPCR melt curve analysis using individual larvae (3–6 dpf). Cas9 injected control (0 ng/µL of sgRNA) was included (*n* = 4 per batch). Data are presented as mean ± SEM with *n* = 3 batches, 10–20 larvae per treatment per batch. Superscripts denote statistically significant difference between sgRNAs.

In tilapia, varied concentrations of gRNAs (50–250 ng/μL) have been used to date^15,21,22^, but there is a lack of data on the optimal ratio of sgRNA to Cas9 to ensure high mutagenesis efficiency and low treatment mortality. We tested three different concentrations of sgRNA (100, 150 and 250 ng/µL) with a constant concentration of Cas9 mRNA (500 ng/µL), with molar ratios of Cas9:sgRNA being 1:9.0, 1:13.4 and 1:22.4, respectively. A single dose of Cas9 was chosen based on previously published results where 500 ng/µL resulted in a higher mutation frequency but a lower survival rate compared to 100 or 300 ng/µL of Cas9 in Nile tilapia^15^. There were no significant differences in treatment mortality in relation to *piwil2* guide RNA design or sgRNA dose (Fig. 1). Mutation frequencies were initially assesed by qPCR melt curve analysis^26^. Mutation frequencies of sgRNA2 were significantly higher than sgRNA1 at all three concentrations tested (Fig. 1). There was no significant difference in mutation frequency between 100, 150 and 250 ng/µL in sgRNA2 embryos (91.6 ± 10.2, 98.2 ± 3 and 97.0 ± 5.2%, respectively). In contrast, sgRNA1 showed highly inefficient mutation frequencies for all sgRNA1 concentrations (1.7 ± 2.9, 5.0 ± 8.7 and 3.3 ± 5.8% for 100, 150 and 250 ng/µL sgRNA1, respectively).

### Impact of piwil2 KO on PGCs survival

**Figure 2:**
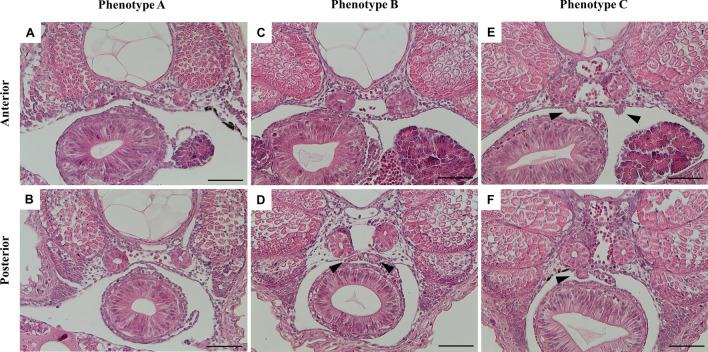
Histological observation of gonadal anlagen and PGCs in serial transverse sections of 3 dah *piwil2* mutants induced by CRISPR/Cas9. Three different phenotypes are shown: (**A**,**B**) type A, no gonadal anlagen and PGCs; (**C**,**D**) type B, morphologically atrophic and/or locally restricted PGCs; and (**E**,**F**) type C, gonadal anlagen and PGCs similar to control. Arrowheads indicate gonadal anlagen. Scale bar = 50 µm.

*piwil2* KO larvae were produced using 150 ng/µL of sgRNA2 and 500 ng/µL of Cas9 mRNAs and its physiological impact on PGCs was investigated through histological observation of PGCs at an early larval stage (pre-first feeding) in Nile tilapia, identified by their location and morphological features^27^. Mutation frequency (initially identified by qPCR melt curve analysis) was 95.8 ± 4.3% and survival rate to 3 days post fertilisation (dpf) (37.8 ± 18.6%) was comparable to uninjected controls (42.5 ± 10.8%). A total of 52 *piwil2* mutant larvae (identified initially by qPCR melt curve analysis) were subjected to histological observation of PGCs using serial transverse sections of the body cavity stained with H&E. As confirmed in uninjected control larvae in the current study (Supplementary Fig. S1), at 3 dah, PGCs were found in the gonadal anlagen located in the dorsal peritoneal wall after the formation of the coelomic cavity in the lateral plate mesoderm, and soon after PGCs started to proliferate^27–29^. The individual mutant phenotypes were subsequently classified based on the histological findings (Fig. 2). There were three apparent phenotypes observed: type A, no gonadal anlagen with putative PGCs observed (15/52, 29%); type B, putative PGCs morphologically atrophic and/or locally restricted (13/52, 25%); and type C, putative PGCs similar to the control (24/52, 46%). As these were all identified as mutants based on initial screening by qPCR melt curve analysis, the diversity in individual mutant genotypes was further studied using NGS.

### Genotyping by NGS

**Figure 3:**
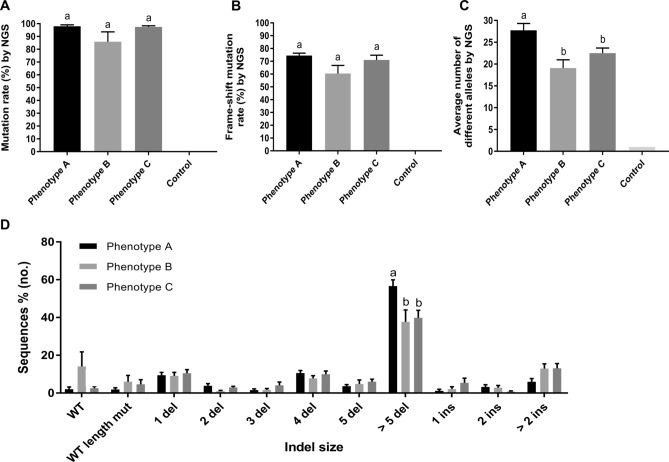
Mutation rates of *piwil2* KO larvae grouped by observed phenotype A, B, and C as well as WT control. (**A**) The average mutation rate assessed by NGS, (**B**) the average frame-shift mutation rate assessed by NGS, (**C**) the average number of different alleles per larva detected by NGS and (**D**) the mean proportion of indel sizes in phenotype A, B and C (*n* = 15, 13 and 24, respectively) assessed by NGS using the representative sequences. Data are shown as mean ± SEM. Superscripts denote a statistically significant difference between groups at each indel size (*p* < 0.05). *WT* wild type sequence, *WT length* WT & WT length mutant, *WT length mut* WT length mutant, *del* deletion, *ins* insertion.

With an average read depth of 10,943 ± 203 per individual analysed using the CRISPResso analysis suite^30^, it was evident that complex mosaic genotypes had been generated in all mutants analysed. The average mutation rates determined by NGS [100 − frequency of WT(%)] were not significantly different between mutant phenotype groups (97.9 ± 1.2, 85.9 ± 7.7 and 97.4 ± 1.0% in phenotypes A, B and C, respectively) (Fig. 3A). Both the frame-shift mutation rates (78.3 ± 1.8, 73.8 ± 4.0 and 74.0 ± 4.1% in phenotype A, B, and C, respectively) (Fig. 3B) and the potential splice site mutation rates (0.5 ± 0.3, 0.3 ± 0.2 and 0.4 ± 0.2%, respectively) were comparable among mutant phenotype groups. The average number of different alleles detected was significantly higher in phenotype A than B and C (28 ± 6, 19 ± 7 and 23 ± 6, respectively) (Fig. 3C). The mean proportion of deletions greater than 5 bp was significantly higher in phenotype A than B and C (Fig. 3D). The most frequent mutant allele was a 4 bp deletion which comprised 8, 7.2 and 8.4% in phenotype A, B and C and putative indels generated by MMEJ made up 25, 16 and 13% of the total indels in each group, respectively (Supplementary Table S2). The frequency of deletion, insertion and substitution events in the 52 mutants was highest at the predicted cleavage position showing 75.5 ± 3.4, 12.6 ± 1.7 and 12.5 ± 1.7%, respectively (Supplementary Fig. S2). Deletion activity in phenotype A was significantly higher than phenotype B at positions 114, 117 and 120–122 while insertion activity in phenotype A was significantly lower than phenotype C at position 122 (Supplementary Fig. S2). There was no apparent difference in substitution activity between any of the phenotypic groups (Supplementary Fig. S2F). Overall, the frequency of 3–19 bp deletion was higher in phenotype A than B and C with the 50th percentile of indel size was 7 bp deletion in phenotype A while being 5 bp deletion in B and C (Supplementary Table S3 and Fig. S3).

### Comparison of mutation screening methods including fragment analysis, T7E1, RGEN and HRMA

**Figure 4:**
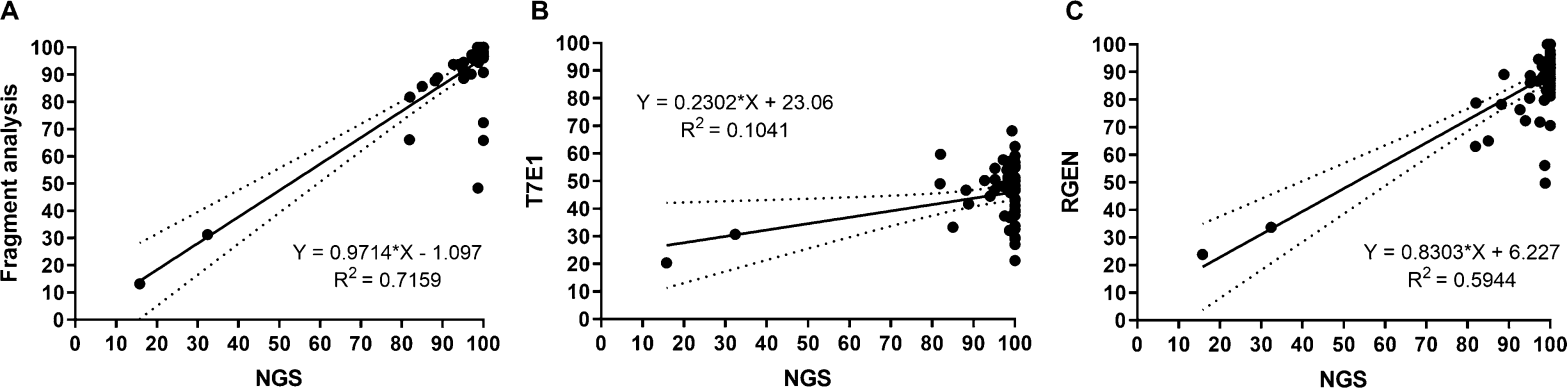
Scatter plot and linear regression between the mutation rate determined by NGS and the arbitrary mutation frequency calculated by (**A**) fragment analysis, (**B**) T7E1 or (**C**) RGEN in 52 mutant samples. Each resulting regression is drawn with its confidence interval at 95% (dotted line).

The genotype of all 52 mutant larvae were further analysed using methods commonly reported in the literature to screen CRISPR/Cas9 efficacy. Regression analysis between mutation rates determined by NGS and the arbitrary gene modification rates assessed by T7E1, RGEN and fragment analysis, revealed a weak correlation with T7E1 (*r*^2^ = 0.10), a moderate correlation with RGEN (*r*^2^ = 0.59) and a high correlation with fragment analysis (*r*^2^ = 0.72) (Fig. 4). Both T7E1 and RGEN are cleavage assays while T7E1 detected mismatches and RGEN detected unmodified alleles^31,32^. Fragment analysis showed that 35 out of 52 mutants had a WT size fragment (Supplementary Fig. S2C) which included both genuine WT and mutant fragments of WT length. The latter was the case for four outliers (Fig. 4A) in which gene modification rate analysed by fragment analysis was underestimated in comparison to NGS due to the presence of 12 to 51.8% of WT length mutant sequences (Supplementary Table S4). In general, the average proportion of indel size assessed by fragment analysis was similar to the NGS result (Supplementary Fig. S5 and Fig. 3D), showing 70.2 ± 2.2% of alleles identified by NGS (*n* = 52) were captured by fragment analysis with a tendency to overlook low abundance fragments (< 2%) (see typical examples in Supplementary Fig. S6). HRM analysis showed that melt curves of all mutants were clearly distinguishable from the control melt curves but there was no clear difference in melting temperature (Tm) between phenotypic groups (Supplementary Fig. S7).

## Discussion

The current study analysed functional impact of *piwil2* KO on PGC development and characterised genotypes of founder Nile tilapia, one of the most cultured aquaculture species worldwide^1^. CRISPR/Cas9 system efficiently edited PIWI domain of *piwil2*, a novel target gene to induce sterility, which is a prerequisite for a direct phenotyping of such sterility-related genes in KO animals. In the current study, there was no significant differences in mutation frequency and mortality in relation to sgRNA concentrations however sgRNA sequence did have a significant impact on the efficacy of the treatment. In zebrafish, increasing concentration of sgRNAs to a constant Cas9 mRNA amount resulted in sgRNA dose-dependent gene editing efficiency^20^. This discrepancy might be caused by the different range of the tested ratios. In the present study, three different molar ratios of Cas9:sgRNA (1:9, 13.4 and 22.4) were above the range tested in zebrafish (i.e. 1:0.3, 1.8 and 9), suggesting that all three ratios tested in this study may, in essence, be at a saturation level, and result in maximum potential mutation frequency. Even though both sgRNAs were designed to target PIWI domain of *piwil2*, there was a significant difference in mutation frequencies between *piwil2* sgRNA1 and sgRNA2. It is possible that the intrinsic properties of the target sites in terms of epigenetic states, transcription activities and high GC-content, could have been the main drivers behind the variable mutation rates observed^20,33,34^. The dramatic difference in mutation efficacy between the two sgRNAs implies there is a caveat in sgRNA designing tools as the criteria for computational prediction of sgRNA efficiency are derived from limited data^35^. Thus, the pragmatic approach to design sgRNA would be to test multiple sgRNAs for each target gene to accommodate for unknown influences of the intrinsic properties of the target regions.

Importantly, mutants in PIWI domain of *piwil2* gene produced by CRISPR/Cas9 showed different degrees of phenotypic impacts on PGC assessed by histological observation, and resulting in more than half of the mutants showing either an apparent absence or morphologically atrophic and locally restricted PGCs in agreement with the suggested role of *piwil2* in the maintenance of PGCs in various species^16,36–39^. Although the histological observation of PGCs at the early larval stage has been widely used in various teleost species^13,40–44^ including Nile tilapia^27–29^, the results based on histological assessment should be interpreted with caution. Clearly, confirmation of phenotype stability (i.e. functional sterility) is required in adult mutants but unfortunately experimental regulation restricted such a confirmation in the present study.

The current study is the first to report deep sequencing data in sterility-related gene functional analysis in a teleost using CRISPR/Cas9 and subsequent NHEJ and MMEJ repairs, and it revealed a high level of mosaicism in F0 animals, with 23 ± 1 different alleles per larva. In addition, in contrast to previous findings showing dominant indels produced by CRISPR/Cas9 were 1 bp insertion (54.1%)^45^, 1–3 bp deletions (49.2%)^46^ or 1 bp deletion (67%)^47^, in this study, 1 bp insertion (3.4%) or 1 and 4 bp deletion (9.8 and 9.6%, respectively) were much less frequent in comparrison to wider indel sizes (i.e. > 5 bp deletion) which correspond to more than 40% of observed mutations. Even though NGS analysis revealed that 26 out of 52 *piwil2* KO larvae (50%) showed 100% biallelic mutation rate, the phenotypic impact was variable. There was no significant difference in the average mutation rates between phenotypic groups which contrasts with previous findings^48–50^ in which a higher degree of mutation appeared to be related to the severity of the phenotypic response. In addition to total mutation rate, the frame-shift mutation rates were investigated as the different proportions of in-frame mutation existing in mutants can generate partial loss-of-function in proteins and impacts on the severity of the phenotype^20^ as shown for *pax2a* edited zebrafish^51^ and *igfbp-2b2* edited rainbow trout^50^. However, frame-shift mutation rates were not apparently correlated with the phenotypes in the present study. No clear correlation between frame-shift mutation rates and the severity of the phenotypes was also reported in red sea bream (*Pagrus major*)^52^. Given that the target area was located towards the 3′ end of *piwil2* (21st exon out of a total of 24 exons), the frame-shift mutation may not make an apparent difference in the phenotype in this case. Since a conserved sequence of PIWI domain was targeted in this study, the phenotypic impact may be more related to the size of deletion at the target site rather than frame-shift rate. This is tentatively supported by a significantly higher proportion of > 5 bp deletions in phenotype A than in B and C which could support a potential link between the severity of mutation effects and the degree of phenotypic response in *piwil2* KO larvae. However, specific genotypic features that can reliably predict the phenotypic response in *piwil2* KO tilapia larvae were not apparent.

The accuracy of the most frequently used mutation screening methods (T7E1, RGEN, HRMA, fragment analysis) were compared to resolve the individual mosaic genotypes observed. Fragment analysis competently detected mutants in F0 animals in a high throughput fashion. This analysis had previously been validated in mutant cell lines generated by CRISPR/Cas9, showing that small indel can be detected with 1 bp resolution and the proportion of mutant fragments can be determined^53,54^. This assay was used in F0 zebrafish to determine editing efficiency of sgRNA and it showed a strong correlation with germline transmission efficiency^35^. As opposed to T7E1, RGENs and HRMA, this analysis was not restricted to the detection of mutants and the estimation of mutation rate, but also provided insight into the diversity and composition of various indel sizes in mutant F0 animals. It was also shown in rainbow trout (*Oncorhynchus mykiss*) that fragment analysis reflected the relative abundance of major indels in F0^50^. Compared to NGS, 70% of indel variants detected by NGS were matched with fragment analysis and the arbitrary gene modification rate calculated by fragment analysis showed the highest correlation with the mutation rate measured by NGS. However, it was noted that the total number of fragments detected by fragment analysis was significantly lower than NGS due to a lower sensitivity and it was unable to detect mutant sequences that had not changed in physical length (i.e. WT length mutated sequences). Therefore, in the context of CRISPR/Cas9 studies, data obtained by fragment analysis are not suitable to assess frame-shift mutation rates or determine the total number of different alleles on target site due to the resolution error of this assay. Overall, fragment analysis offers an overview of the indel size variants, but the wide application of this method in mutant screening is hampered by the inability to provide sequence information.

Although T7E1 and RGENs were easy to operate, they cannot be easily applied for large-scale screening. In addition, the mutation frequencies detected by T7E1 were consistently lower than the mutation rate determined by NGS in accordance with previous reports^31,55^ due to the inefficient heteroduplex formation, overlooking of homoduplexes for the mutation rate estimation and possible incomplete digestion of nucleases^32^. Being based on enzyme reactions, incomplete digestion due to suboptimal reaction conditions can cause false negative results in T7E1 assay or false positive results in RGENs. While HRMA detected the existence of mutation, it was unable to provide further information on genotypes of *piwil2* KO F0 generated by CRISPR/Cas9. It was also shown in F0 zebrafish induced by CRISPR/Cas9, TALEN or ZFN that HRMA is rapid and capable of high throughput screening for mutation detection^25,56–58^, but no further genotypic information could be obtained. Therefore, melt curve analyses could be used as an initial screening for F0 generation prior to sequencing. All methods tested here reliably identified the mutants, but T7E1, RGENs and HRMA could not clarify the complexity of the mosaic genotypes. Fragment analysis could capture and map indel size spectrum and the estimated mutation rate was the closest to the actual mutation rate analysed by NGS among all methods tested. However, the limited resolution of size for detection and the lack of sequence details hinder the clear depiction of the mutation diversity in mutants. Certainly, NGS was the most informative, accurate and high throughput screening method of all five methods tested. As demonstrated in this study it is possible to access the power of NGS genotyping in a cost-effective manner if low volume sequencing libraries can be multiplexed into other sequencing experiments. It is therefore essential that going forward in order to understand and resolve the complexity of F0 genotypes, NGS sequencing should be used as a suitable methodological approach^50,59^.

In summary, the functional importance of PIWI domain of *piwil2* gene on PGCs was revealed in Nile tilapia, showing that *piwil2* KO can result in PGC deficient phenotypes at the early larval stage. Among various mutant detection methods NGS was the most informative and reliable assay to genotype the KO individuals. The deep sequencing analysis of the resultant genotypes suggested that while the total mutation and frame-shift mutation rates did not clearly correlate with the observed phenotypes, the indel size distribution showed that the PGC deficient phenotype had significantly higher proportion of > 5 bp large deletions. However, high mosaicism and wide indel spectrum prevented the elucidation of a clear link between genotype and phenotype. Thus, future studies should focus on reducing mosaicism which could include usage of Cas ribonucleoproteins and machine learning models to predict genotype of gRNAs and editing of germline cells^60,61^. This will facilitate direct functional analysis in F0.

## Methods

### *Handling of gametes, *in vitro* fertilisation and microinjection*

Zygotes were produced from mature female (XX) and supermale (YY) tilapia (*O. niloticus* L.) held in the tropical aquarium of the Institute of Aquaculture at the University of Stirling. Eggs were washed with water from the aquarium system and kept at 25 ± 1 °C for up to 2.5 h (hrs) before fertilisation. The milt was collected by glass capillary and stored in a sterile test tube over ice for up to 2.5 h before fertilisation. Approximately 400–500 eggs were fertilised in a single Petri dish by adding 4 µL of milt and activating the milt by adding fresh aquarium water. The fertilised eggs were rinsed 3 min after fertilisation. The fertilised eggs were kept at 21 ± 1 °C to extend the 1 cell stage for up to 2.5 h^62^. Each 1 cell stage embryo was held by the holding pipette and the Cas9 RNA and sgRNAs mixture was injected into the embryonic cell. The control and injected eggs were incubated according to standard practice for tilapia in round bottom recirculating tanks at 27 ± 1 °C under 12L:12D photoperiod.

### Design and off-target search for piwil2 sgRNAs

Two sgRNAs were designed using CRISPR RGEN tool (https://www.rgenome.net/) and Benchling (https://benchling.com) and the potential off-target sites were checked by in silico off-target analysis using NCBI BLAST (https://blast.ncbi.nlm.nih.gov/Blast.cgi) and Cas-OFFinder (https://www.rgenome.net/cas-offinder/). Any sgRNAs containing potential off-targets which had more than 12 nt identical to the seed sequences adjacent to the protospacer adjacent motif (PAM), out of the 20 nt sgRNAs, were excluded^63,64^ (Supplementary Table S1). Two sgRNAs were selected which were located in exon 24 (sgRNA1) and 21 (sgRNA2) on the conserved domain of PIWI.

### Preparation of sgRNAs and Cas9 mRNAs

sgRNA template was produced by a PCR approach^65^ using pT7-gRNA plasmid as sgRNA scaffold template, which was supplied by Wenbiao Chen (Addgene plasmid #46759)^20^ and gRNA specific primer added with a T7 promoter sequence (Table 1). The purified PCR product was subsequently used as template for RNA synthesis using HiScribe T7 High Yield RNA Synthesis Kit (NEB). Then, they were purified using RNeasy Mini Kit (Qiagen) and quantified by spectrophotometry (NanoDrop). The size and integrity of purified sgRNAs were checked by gel electrophoresis with Low Range ssRNA ladder (NEB) before storage at − 20 °C until use. Cas9 mRNAs (*S. pyogenes*) were prepared using pT3TS-nCas9n, supplied from Wenbiao Chen (Addgene plasmid #46757)^20^. The Cas9 ORF template was amplified by PCR and the purified PCR product was subsequently used as a template for RNA synthesis using mMESSAGE mMACHINE T3 Transcription kit (Thermo Fisher). The transcribed capped Cas9 mRNA were purified using RNeasy Mini Kit (Qiagen) and quantified by spectrophotometer. The size and integrity of purified Cas9 mRNA were checked by gel electrophoresis with ssRNA ladder (NEB) before storage at − 20 °C until use.

**Table 1:** Primer list used for *piwil2* sgRNA production, screening of *piwil2* mutants.

Primer pair	Forward primer (5′–3′)	Reverse primer (5′-3′)	Annealing temp (°C)	Purpose
sgRNA scaffold	GTTTTAGAGCTAGAAATAGCAAG	AAAAGCACCGACTCGGTG	53.7	Template of sgRNA scaffold
*piwil2* T7 sgRNA1	*GATCACTAATACGACTCACTATAGGGCTGGAACACGAATGGTGCC*GTTTTAGAGCTAGAAAT	AAAAGCACCGACTCGGTGCCACTTTTTCAAGTTGATAAC	71.6	Template of *piwil2* sgRNA1
*piwil2* T7 sgRNA2	*GATCACTAATACGACTCACTATAGGACGGATCAGTTCCTCATTGG*GTTTTAGAGCTAGAAAT	AAAAGCACCGACTCGGTGCCACTTTTTCAAGTTGATAAC	70.9	Template of *piwil2* sgRNA2
M13 universal	GTAAAACGACGGCCAGT	AACAGCTATGACCATG	58	To amplify ORF of Cas9 from pT3TS-nCas9n
*piwil2* sgRNA1	AACAGGTAACTGCTGTCTGCAT	TTGGTTTCTTGCCAGGTTGACTT	56.5	qPCR melt curve analysis
*piwil2* sgRNA2	TAGGTGAGAATTAGGTGTGGTTT	TGCACAATGCATGAGTCCTAC	55.5	qPCR melt curve analysis, HRMA,
*piwil2* gRNA2_2	ACCTGTGCCGTAAGGCTGGA	AGTGTGCAGAAAACACTGACTTCAC	67.5	T7E1 assay, RGEN assay
*piwil2* gRNA2_ CAG	**cagtcgggcgtcatca**TAGGTGAGAATTAGGTGTGGTTT	TGCACAATGCATGAGTCCTAC	56.7	Fragment analysis
*piwil2* gRNA2 NGS	TCGTCGGCAGCGTCAGATGTGTATAAGAGACAGTAGGTGAGAATTAGGTGTGGTT*T	GTCTCGTGGGCTCGGAGATGTGTATAAGAGACAGTGCACAATGCATGAGTCCTA*C	55.5	NGS

Italic, T7 promoter; underline, target sequence of Nile tilapia *piwil2*; bold lowercase, CAG tailing sequence; boxed sequences, Illumina overhang adapter sequences. The primer pair for HRMA and NGS were purified by HPLC. NGS primer pair has 3′ modification. Asterix (*) denotes a phosphorothioate (PTO) bond.

### Microinjection of different ratios of Cas9 and sgRNA

Three different sgRNA concentrations (100, 150 and 250 ng/µL) combined with a single concentration of Cas9 (500 ng/µL) were tested for each sgRNA. Data were collected from three independent Nile tilapia egg batches for a given molar ratio. The total injected embryo numbers were 530, 381 and 422 for 100, 150 and 250 ng/µL of sgRNA1, respectively, and 405, 368 and 456 for 100, 150 and 250 ng/µL of sgRNA2, respectively (total number was divided between three independent egg batches for each concentration of sgRNA). Each egg batch included two control sub-groups: a non-injected control and a 500 ng/µL Cas9 injected control (*n* = 2,377 and 786, respectively). The survival rate was recorded at 3 dpf and the treatment mortality was calculated against the mortality rate of control using the Schneider–Orelli’s formula. The larvae from the injected group and controls were sampled before the first feeding stage (3 dah) after euthanasia using 60 ppm benzocaine for subsequent analysis.

### gDNA extraction and qPCR melt curve analysis

gDNAs from individual larvae were extracted by lysing at 55 °C using 200 μL of SSTNE (0.5 mM spermidine, 0.15 mM spermine, 50 mM Tris, 0.3 M NaCl and 0.2 mM EGTA) buffer, 20 μL of 10% SDS (sodium dodecyl sulphate-anionic detergent) and 5 μL of Proteinase K (10 mg/μL) followed by 5 μL of RNase A (2 mg/μL) treatment and salt precipitation. Briefly, 161 µL of 5 M NaCl was added and centrifuged to precipitate proteins. Then, 250 μL of each supernatant was collected and the same volume of isopropanol was used to precipitate gDNA. gDNA pellets were washed twice with 75% ethanol and resuspended in 10–20 μL of 5 mM Tris (pH 8.0) depending on the size of the pellet. Two primer pairs were designed to amplify DNA fragment including target area of *piwil2* sgRNA1 and sgRNA2 and used for qPCR melt curve analysis^26^ (Table 1). Each qPCR reaction was of a total volume of 10 μL containing 1 μL of gDNA (70–350 ng), 5 μL of SYBR green mix (Luminaris Color HiGreen qPCR Master Mix, Thermo Fisher), 0.7 μM of each forward and reverse primer and MiliQ water up to 10 μL. Mastercycler ep realplex (Eppendorf) was used and the qPCR thermal cycling protocol was: 50 °C for 2 min, 95 °C for 10 min, followed by 40 cycles of 95 °C for 15 s, 56.5 (sgRNA1) or 55.5 °C (sgRNA2) for 15 s and 72 °C for 20 s. It was followed by melt curve analysis of 95 °C for 15 s, 60 °C for 15 s, a ramp increment at 0.023 °C/s from 60 to 95 °C with a continuous fluorescence detection and 95 °C for 15 s. All samples were analysed in duplicate together with non-template controls. The total number of the injected larvae subjected to mutant screening was 60 (20, 20, 20), 52 (12, 20, 20) and 49 (10, 19, 20) for 100, 150 and 250 ng/µL of sgRNA1 and 59 (20, 20, 19), 50 (19, 11, 20) and 51 (20, 11, 20) for 100, 150 and 250 ng/µL of sgRNA2, respectively (three batches for each concentration of sgRNA). The number of both Cas9 injected control (0 ng/µL of sgRNA) and uninjected control subjected to mutant screening was four per treatment per batch. Presence of mutations in the target site of each sample was assessed by the shape of its derivative of fluorescence with respect to the temperature (dF/dT) dissociation curves using Mastercycler ep realplex (Eppendorf) Software.

### H&E staining for histological observation of PGCs in piwil2 KO individuals

The fixed tissues were dehydrated in an ascending ethanol series, cleared in xylene and then infiltrated with paraffin wax. Every trunk tissue was embedded in paraffin with an anterior–posterior presentation for transverse section using a histoembedder and sectioned using a Rotary microtome. Based on the preliminary screening of PGCs to locate precisely the gonadal anlagen in 3 dah control larvae (*n* = 5), the histological analysis of the *piwil2* sgRNA2/Cas9 injected larvae was conducted in the regions A-D where PGCs were reliably detected using the serial transverse section of body cavity stained with H&E (Supplementary Fig. S1). The six consecutive serial sections at 5 µm thickness collected at every 150 µm interval in the area from A to D were analysed for every sample and the presence of PGCs with its location and appearance was recorded in the confirmed 52 *piwil2* mutant larvae by qPCR melt curve analysis.

### NGS

Sequencing libraries of the target region of *piwil2* sgRNA2 from both control and putative mutants gDNA samples were prepared according to the Illumina MiSeq system instructions. The target region was amplified and indexed by 20 cycle and 10 cycle of PCR, respectively. Each amplicon was purified using AxyPrep Mag PCR Clean-up Kit (Axygen), quantified by using the Qubit 2.0 Fluorometer and dsDNA HS Assay Kit (Thermo Fisher) and added into the library at the final concentration of 1 nM. The library was quantified by using the Qubit 2.0 Fluorometer and dsDNA HS Assay Kit (Thermo Fisher) and normalised to the final concentration of 10 nM. The double-indexed library was combined with an existing MiSeq sequencing run. Libraries were sequenced on an Illumina MiSeq instrument using a MiSeq Reagent Kit V2 of 250 bp paired-end reads with the *piwil2* CRISPR library representing 2.5% of the total sequencing run, along with a 6% phiX library (control). In total 20.5 M paired-end reads were produced of which 0.66 M paired-end reads belonged to the CRISPR study with the average number of reads per sample being 10,943 ± 203 per sample. FASTQ files generated by Illumina sequencing were analysed with CRISPResso^30^. The analysis settings of CRISPResso were as follow: (1) minimum average read quality, > 99.9% confidence (phred33 ≥ 30) per read; minimum single base pair quality, > 90% confidence (phred33 ≥ 10) per base pair, (2) mutation was quantified within a window of 81 bp upstream and 70 bp downstream from the canonical cleavage site, between third and fourth nucleotide upstream of preceding PAM sequence. Then the trimmed reads were merged to be paired-end sequences, aligned to a reference amplicon and the proportion of indel was quantified^30^. Each mutation rate (%) was calculated by substracting the percentage of NGS reads of WT (%) from 100 (%). In addition, frame-shift mutation rate (%) was calculated by dividing frame-shift mutation reads (No.) by ∑total reads (No.) and multiplying by 100. Sequences with MMEJ-mediated repair were predicted by Microhomology-Predictor (https://www.rgenome.net/mich-calculator/)^66^.

### T7 endonuclease 1 (T7E1)

Prior to T7E1 digestion, 100 ng of each purified amplicon was hybridised to form heteroduplex in 10 μL reaction volume containing 1 μL of NEBuffer 2 (NEB). The thermocycler condition for hybridisation was as follows: initial denaturation at 95 °C for 5 min, annealing from 95 °C to 85 °C at − 2 °C/s ramp rate and 85 °C to 25 °C at − 0.1 °C/s ramp rate and termination at 20 °C. The hybridised product was digested by 0.5 μL of T7EI (NEB) at 37 °C for 15 min and terminated by adding 1 μL of 0.25 M EDTA. The fragmented PCR products were then run on the agarose gel and the percent of nuclease-specific cleavage products were determined by GeneTools (Syngene). The cleavage efficiency of T7E1 was calculated^67^ and used as the estimated arbitrary gene modification rate. The intra- and inter-assay CVs were 1.98 and 3.95%, respectively.

### CRISPR/Cas-derived RNA-guided engineered nuclease (RGEN) assay

The *piwil2* sgRNA2 and Cas9 nuclease protein (*S. pyogenes*) (NEB) were used to examine the mutation efficiency of *piwil2* sgRNA2 mutants, according to the manufacturer’s protocol. 50 nM of *piwil2* sgRNA2 and 50 nM of Cas9 nuclease were incubated at 25 °C for 10 min to assemble the Cas9/sgRNA complex. Then, the purified amplicons were added at the final concentration of 4.5 nM as the substrate DNAs and incubated at 37 °C for 16 h. The assay included eight positive controls of WT samples and three negative controls of no DNA substrates. Molar ratio of Cas9 and sgRNA per target site was maintained at 11:11:1 in a total reaction volume of 10 μL. The cleavage reaction was terminated by incubating at 80 °C for 5 min. The fragmented PCR products were then resolved with 1% agarose gel electrophoresis. The proportion of nuclease-specific cleavage products were determined by measuring each band intensity using GeneTools (Syngene) to allow the estimation of arbitrary gene modification rate^31^. The intra- and inter-assay CVs were 1.96 and 3.50%, respectively.

### HRMA

The PCR reaction for HRMA contained 5 μL of 2X LightCycler 480 High Resolution Melting Master Mix (Roche), 1.2 μL of 25 mM MgCl2, 0.3 μL each 10 μM primer (Table 1), 25 ng of gDNA and Milli-Q water up to 10 μL. Each mutant and control sample was tested in triplicate. The PCR program was: pre-incubation at 95 °C for 10 min, 45 cycles of denaturation at 95 °C for 15 s, touchdown annealing (62 °C to 57 °C with 0.5 °C decrement/cycle) for 15 s and extension at 72 °C for 15 s. Followed by HRMA program: 95 °C for 1 min, 40 °C for 1 min, 70 °C to 95 °C with 25 acquisitions per degree centigrade. The result was analysed by Gene Scanning and Tm calling analyses in LightCycler 480 Software. The intra- and inter-assay CVs of melt temperature (Tm) were 0.02 and 0.08%, respectively.

### Fragment analysis

PCR was performed using a fluorescent labelled tailed primer method^68^. In this study, CAG_green (5′Dye-CAGTCGGGCGTCATCA-3′) (Sigma-Aldrich) was used to detect the mutations created by *piwil2* sgRNA2. This dye sequence was added to the 5′ prime end of the forward primer and paired with non-tailed reverse primer (Table 1). The PCR reactions (8 µL total volume) consisted of 4 μL of Q5 Hot Start High-Fidelity 2X Master Mix (NEB), 0.15 μL of 1 μM tailed forward primer, 0.25 μL of 10 μM non-tailed reverse primer and 0.25 μL of 10 μM fluorescent dye labelled primer, 25 ng of gDNA and Milli-Q water. PCR program was: 98 °C for 30 s, followed by 33 cycles of 98 °C for 10 s, 62 °C for 20 s and 72 °C for 20 s, with a final extension at 72 °C for 2 min. The size determination was performed using a Beckman Coulter CEQ8000 Sequencer (Beckman Coulter). All the obtained fragment lengths from the module were standardised based on the WT fragment length in controls of 227.69 ± 0.04 nt (*n* = 8) with indel size thereafter being described as ± WT length with the indel size values being rounded off to the nearest whole nucleotide number. The proportion of each fragment within the mosaic genotype was calculated based on the height of the fragment^35^. The estimated arbitrary gene modification rate (%) assessed by fragment analysis was calculated by substracting the proportional height of zero indel fragment (%) from 100 (%).

### Statistics

Statistical analysis was performed using Minitab 17 (Minitab Inc.). Data are presented as mean ± SEM. Significant differences between group mean were tested by one-way ANOVA, followed by Tukey’s HSD test (*p* < 0.05) for arbitrary gene modification rate by T7E1, number of different size fragments detected by fragment analysis, frame-shift mutation rate, number of different alleles and different size fragment analysed by NGS, proportion of indel size between phenotype groups analysed by fragment analysis and NGS, detected fragment number between fragment analysis and NGS, positional differences in deletion, insertion or substitution frequencies between the different phenotype groups around the canonical cut site. All percentage data were arcsine transformed and normality and homogeneity of variance were confirmed through examination of the model residuals and fits and Levene’s test, and where necessary data was further square root or log10 transformed.

When control samples were all registered as 0 or 1 (arbitrary gene modification rate by T7E1, and mutation rate, frame-shift mutation rate, number of different alleles and different size fragment analysed by NGS), they were excluded from statistical analysis. Significant differences between group mean which did not meet the normality of variance for arbitrary gene modification rates by RGEN and fragment analysis and mutation rate by NGS were assessed by Kruskal–Wallis test, followed by Mann–Whitney test (*p* < 0.05). The linear regression was performed by using GraphPad Prism v7.03 (GraphPad Software) between the mutation rate determined by NGS and the arbitrary gene modification rates calculated by T7E1, RGEN and Fragment analyses.

### Ethics statement

All working procedures were carried out in accordance with the United Kingdom Animals (Scientific Procedures) Act 1986 and were approved by the ethics committee and the GM committee of the University of Stirling.

## Supplementary information

Supplementary Information.

Supplementary Figure S1.

Supplementary Figure S2.

Supplementary Figure S3.

Supplementary Figure S4.

Supplementary Figure S5.

Supplementary Figure S6.

Supplementary Figure S7.

## Data Availability

All relevant data are within the paper and its Supporting Information files.

## References

[1] FAO (2019). FAO Yearbook. Fishery and Aquaculture Statistics 2017.

[2] Belton B, Bush SR, Little DC (2018). Not just for the wealthy: Rethinking farmed fish consumption in the Global South. Glob. Food Security.

[3] Hulata G, Wohlfarth G, Rothbard S (1983). Progeny-testing selection of tilapia broodstocks producing all-male hybrid progenies—Preliminary results. Aquaculture.

[4] Phelps RP, Popma TJ, Costa-Pierce BA, Rakocy JE (2000). Sex reversal of tilapia. Tilapia Aquaculture in the Americas.

[5] Arthur RI (2010). Assessing impacts of introduced aquaculture species on native fish communities: Nile tilapia and major carps in SE Asian freshwaters. Aquaculture.

[6] Canonico GC, Arthington A, Mccrary JK, Thieme ML (2005). The effects of introduced tilapias on native biodiversity. Aquat. Conserv. Mar. Freshw. Ecosyst..

[7] Sun A (2015). Establishment and characterization of a gonad cell line from half-smooth tongue sole *Cynoglossus semilaevis* pseudomale. Fish Physiol. Biochem..

[8] Xie Q-P (2016). Haploinsufficiency of SF-1 causes female to male sex reversal in Nile Tilapia *Oreochromis niloticus*. Endocrinology.

[9] Zhang X (2014). Isolation of *doublesex-* and *mab-3*-related transcription factor 6 and its involvement in spermatogenesis in tilapia. Biol. Reprod..

[10] Li M (2015). A tandem duplicate of anti-müllerian hormone with a missense SNP on the Y chromosome is essential for male sex determination in Nile Tilapia *Oreochromis niloticus*. PLOS Genet..

[11] Jiang D (2017). CRISPR/Cas9-induced disruption of *wt1a* and *wt1b* reveals their different roles in kidney and gonad development in Nile tilapia. Dev. Biol..

[12] Wargelius A (2016). *Dnd* knockout ablates germ cells and demonstrates germ cell independent sex differentiation in Atlantic salmon. Sci. Rep..

[13] Sawamura R, Osafune N, Murakami T, Furukawa F, Kitano T (2017). Generation of biallelic F0 mutants in medaka using the CRISPR/Cas9 system. Genes Cells.

[14] Jin YH, Davie A, Migaud H (2019). Expression pattern of *nanos, piwil, dnd, vasa* and *pum* genes during ontogenic development in Nile tilapia *Oreochromis niloticus*. Gene.

[15] Li M (2014). Efficient and heritable gene targeting in tilapia by CRISPR/Cas9. Genetics.

[16] Kuramochi-Miyagawa S (2004). *Mili*, a mammalian member of *piwi* family gene, is essential for spermatogenesis. Development.

[17] Simon B (2011). Recognition of 2’-O-methylated 3’-end of piRNA by the PAZ domain of a Piwi protein. Structure.

[18] Liu J (2004). Argonaute2 is the catalytic engine of mammalian RNAi. Science.

[19] Xiang G, Zhang X, An C, Cheng C, Wang H (2017). Temperature effect on CRISPR-Cas9 mediated genome editing. J. Genet. Genom..

[20] Jao L-E, Wente SR, Chen W (2013). Efficient multiplex biallelic zebrafish genome editing using a CRISPR nuclease system. Proc. Natl. Acad. Sci..

[21] Chen J (2017). Heterozygous mutation of eEF1A1b resulted in spermatogenesis arrest and infertility in male tilapia *Oreochromis niloticus*. Sci. Rep..

[22] Feng R (2015). Retinoic acid homeostasis through *aldh1a2* and *cyp26a1* mediates meiotic entry in Nile tilapia (*Oreochromis niloticus*). Sci. Rep..

[23] Jiang D-N (2016). *gsdf* is a downstream gene of *dmrt1* that functions in the male sex determination pathway of the Nile tilapia. Mol. Reprod. Dev..

[24] Qin Z (2016). Editing of the luteinizing hormone gene to sterilize channel catfish, *Ictalurus punctatus*, using a modified zinc finger nuclease technology with electroporation. Mar. Biotechnol..

[25] Zhang Z, Lau S-W, Zhang L, Ge W (2015). Disruption of zebrafish follicle-stimulating hormone receptor (*fshr*) but not luteinizing hormone receptor (*lhcgr*) gene by TALEN leads to failed follicle activation in females followed by sexual reversal to males. Endocrinology.

[26] D’Agostino Y (2016). A rapid and cheap methodology for CRISPR/Cas9 Zebrafish mutant screening. Mol. Biotechnol..

[27] Ijiri S (2008). Sexual dimorphic expression of genes in gonads during early differentiation of a Teleost Fish, the Nile Tilapia *Oreochromis niloticus*. Biol. Reprod..

[28] Farlora R (2014). Intraperitoneal germ cell transplantation in the Nile tilapia *Oreochromis niloticus*. Mar. Biotechnol. (NY).

[29] Kobayashi T, Kajiura-Kobayashi H, Nagahama Y (2000). Differential expression of *vasa* homologue gene in the germ cells during oogenesis and spermatogenesis in a teleost fish, tilapia *Oreochromis niloticus*. Mech. Dev..

[30] Pinello L (2016). Analyzing CRISPR genome-editing experiments with CRISPResso. Nat. Biotechnol..

[31] Kim JM, Kim D, Kim S, Kim J-S (2014). Genotyping with CRISPR-Cas-derived RNA-guided endonucleases. Nat. Commun..

[32] Zischewski J, Fischer R, Bortesi L (2017). Detection of on-target and off-target mutations generated by CRISPR/Cas9 and other sequence-specific nucleases. Biotechnol. Adv..

[33] Doench JG (2016). Optimized sgRNA design to maximize activity and minimize off-target effects of CRISPR-Cas9. Nat. Biotechnol..

[34] Wu X (2014). Genome-wide binding of the CRISPR endonuclease Cas9 in mammalian cells. Nat. Biotechnol..

[35] Carrington B, Varshney GK, Burgess SM, Sood R (2015). CRISPR-STAT: an easy and reliable PCR-based method to evaluate target-specific sgRNA activity. Nucleic Acids Res..

[36] Houwing S, Berezikov E, Ketting RF (2008). Zili is required for germ cell differentiation and meiosis in zebrafish. EMBO J..

[37] Houwing S (2007). A role for Piwi and piRNAs in germ cell maintenance and transposon silencing in Zebrafish. Cell.

[38] Zhao H, Duan J, Cheng N, Nagahama Y (2012). Specific expression of *Olpiwi1* and *Olpiwi2* in medaka (*Oryzias latipes*) germ cells. Biochem. Biophys. Res. Commun..

[39] Li M, Hong N, Gui J, Hong Y (2012). Medaka *piwi* is essential for primordial germ cell migration. Curr. Mol. Med..

[40] Boonanuntanasarn S (2016). Characterization of a *vasa* homolog in the brown-marbled grouper (*Epinephelus fuscoguttatus*) and its expression in gonad and germ cells during larval development. Fish Physiol. Biochem..

[41] Fernández JA (2015). Primordial germ cell migration in the yellowtail kingfish (*Seriola lalandi*) and identification of stromal cell-derived factor 1. Gen. Comp. Endocrinol..

[42] Goto R (2012). Germ cells are not the primary factor for sexual fate determination in goldfish. Dev. Biol..

[43] Hamasaki M (2017). Production of tiger puffer *Takifugu rubripes* offspring from triploid grass puffer *Takifugu niphobles* parents. Mar. Biotechnol..

[44] Hashimoto Y (2004). Localized maternal factors are required for zebrafish germ cell formation. Dev. Biol..

[45] Ma X (2015). A robust CRISPR/Cas9 system for convenient, high-efficiency multiplex genome editing in monocot and dicot plants. Mol. Plant.

[46] Pan C (2016). CRISPR/Cas9-mediated efficient and heritable targeted mutagenesis in tomato plants in the first and later generations. Sci. Rep..

[47] Seeger C, Sohn JA (2016). Complete Spectrum of CRISPR/Cas9-induced Mutations on HBV cccDNA. Mol. Ther..

[48] Edvardsen RB, Leininger S, Kleppe L, Skaftnesmo KO, Wargelius A (2014). Targeted mutagenesis in Atlantic salmon (*Salmo salar* L.) using the CRISPR/Cas9 system induces complete knockout individuals in the F0 generation. PLoS ONE.

[49] Shigeta M (2016). Rapid and efficient analysis of gene function using CRISPR-Cas9 in *Xenopus tropicalis* founders. Genes Cells.

[50] Cleveland BM, Yamaguchi G, Radler LM, Shimizu M (2018). Editing the duplicated insulin-like growth factor binding protein-2b gene in rainbow trout (*Oncorhynchus mykiss*). Sci. Rep..

[51] Trubiroha A (2018). A rapid CRISPR/Cas-based mutagenesis assay in Zebrafish for identification of genes involved in thyroid morphogenesis and function. Sci. Rep..

[52] Kishimoto K (2018). Production of a breed of red sea bream *Pagrus major* with an increase of skeletal muscle mass and reduced body length by genome editing with CRISPR/Cas9. Aquaculture.

[53] Ramlee MK, Yan T, Cheung AMS, Chuah CTH, Li S (2015). High-throughput genotyping of CRISPR/Cas9-mediated mutants using fluorescent PCR-capillary gel electrophoresis. Sci. Rep..

[54] Yang Z (2015). Fast and sensitive detection of indels induced by precise gene targeting. Nucleic Acids Res..

[55] Sentmanat MF, Peters ST, Florian CP, Connelly JP, Pruett-Miller SM (2018). A survey of validation strategies for CRISPR-Cas9 editing. Sci. Rep..

[56] Dahlem TJ (2012). Simple methods for generating and detecting locus-specific mutations induced with TALENs in the Zebrafish Genome. PLoS Genet..

[57] Samarut É, Lissouba A, Drapeau P (2016). A simplified method for identifying early CRISPR-induced indels in zebrafish embryos using High Resolution Melting analysis. BMC Genom..

[58] Thomas HR, Percival SM, Yoder BK, Parant JM (2014). High-throughput genome editing and phenotyping facilitated by high resolution melting curve analysis. PLoS ONE.

[59] Straume AH (2020). Indel locations are determined by template polarity in highly efficient in vivo CRISPR/Cas9-mediated HDR in Atlantic salmon. Sci. Rep..

[60] Mehravar M, Shirazi A, Nazari M, Banan M (2019). Mosaicism in CRISPR/Cas9-mediated genome editing. Dev. Biol..

[61] Shen MW (2018). Predictable and precise template-free CRISPR editing of pathogenic variants. Nature.

[62] Rahman MA, Maclean N (1992). Production of transgenic tilapia (*Oreochromis niloticus*) by one-cell-stage microinjection. Aquaculture.

[63] Cong L (2013). Multiplex genome engineering using CRISPR/Cas systems. Science.

[64] Jinek M (2012). A programmable dual-RNA-guided DNA endonuclease in adaptive bacterial immunity. Science (80-)..

[65] Shao Y (2014). CRISPR/Cas-mediated genome editing in the rat via direct injection of one-cell embryos. Nat. Protoc..

[66] Bae S, Kweon J, Kim HS, Kim J-S (2014). Microhomology-based choice of Cas9 nuclease target sites. Nat. Methods.

[67] Guschin DY (2010). A rapid and general assay for monitoring endogenous gene modification. Methods Mol. Biol. Clifton NJ.

[68] Boutin-Ganache I, Raposo M, Raymond M, Deschepper CF (2001). M13-tailed primers improve the readability and usability of microsatellite analyses performed with two different allele-sizing methods. Biotechniques.

