# Supplementary material for: Physiological impact and comparison of mutant screening methods in piwil2 KO founder Nile tilapia produced by CRISPR/Cas9 system
Source: Sci Rep. 2020 Jul 28;10:12600. doi: 10.1038/s41598-020-69421-0 (PMC7387559; doi:10.1038/s41598-020-69421-0)
Supplement: Supplementary file 1 — Supplementary Information. [file 41598_2020_69421_MOESM1_ESM.docx]

**Physiological impact and comparison of mutant screening methods in *piwil2* KO founder Nile tilapia produced by CRISPR/Cas9 system**

**Ye Hwa Jin^1,2^, Baoshan Liao^1,3^, Herve Migaud^1^ & Andrew Davie^1,*^**

**SUPPLEMENTARY INFORMATION**

**SUPPLEMENTARY FIGURE LEGENDS**

**Supplementary Figure S1.** Histological observation of gonadal anlagen and PGCs in serial transverse sections of 3 dah control larva. (A-E) Transverse view of the body cavity (anterior-posterior) stained with H&E. The position of each histology section corresponding to images A to E are illustrated by the black dotted lines and the interval between the dotted lines is 150 µm. The embedded trunk tissues of 3 dah control larvae were approx. 2.3 mm in length (indicated by two green lines in the lateral view of 3 dah larva). The red shaded box indicates the location of putative gonadal anlagen. NTC, remnants of the notochord; C, centrum; N, pronephric tubules; G, glomeruli; P, parietal peritoneum; M, mesenteries; Pn, pancreas; G, gut. Arrowheads indicate gonadal anlagen. Scale bar = 50 µm (A-E).

**Supplementary Figure S2**. Mutation position distribution. (A-B) deletion, (C-D) insertion, and (E-F) substitution. (A, C & E) the average of all 52 mutants and (B, D &F) the average of phenotype group A (*n* = 15), B (*n* = 13), and C (*n* = 24). The predicted cleavage position is indicated by dotted line at 121 bp in total 211 bp amplicon. Data are shown as mean ± SEM. Superscripts denote statistically significant difference between phenotype groups within the same position (bp) (*p* < 0.05).

**Supplementary Figure S3.** Frequency of indel size in each phenotype. (A) Each dot indicates the frequency of each indel size in each mutant phenotype groups. For example, 100% of -11 bp in phenotype A means all mutants in group A possess -11 bp of indel size regardless of reads % of the indel size of -11 bp in each individual. (B) the box shows 25^th^, 50^th^ and 75^th^ percentiles and whiskers show 5^th^ and 95^th^ percentiles. Indel sizes located outside of 5 – 95^th^ percentile are shown as dots.

**Supplementary Figure S4.** Representative results of fragment analysis. (A) WT control, (B) *piwil2* mutant which does not possess WT length fragment and (C) *piwil2* mutant which possesses WT length fragment. WT length fragment is denoted by asterisk (*). The peaks of size marker are present at 200, 220 and 240 bp with red line.

**Supplementary Figure S5.** Average proportion of indel size in phenotype A, B and C mutants (*n* = 15, 13 and 24, respectively) assessed by fragment analysis using the representative sequences. Data are shown as mean ± SEM. Superscripts denote statistically significant difference between phenotypic groups at each indel size (*p* < 0.05). WT, wild type sequence; WT length, WT & WT length mutant; WT length mut, WT length mutant; del, deletion; ins, insertion.

**Supplementary Figure S6.** Representative results of detected fragments by NGS and fragment analysis. (A) *piwil2* mutant which does not possess WT length fragment (mutant 11, phenotype A) and (B) *piwil2* mutant which has WT length fragment (mutant 21, phenotype B). WT length fragment is denoted by asterisk (*).

**Supplementary Figure S7.** HRMA of *piwil2* mutants and control. (A) Melt curves of WT control (blue, *n* = 8) and mutant phenotype A (red, *n* = 15), B (green, *n* = 13) and C (purple, *n* = 24). (B-E) Distribution and frequency of Tm peaks as identified by HRMA in WT control, phenotype A, B and C.

**Supplementary Table S1**. Potential off-target sites for *piwil2* sgRNAs used in this study

| **Bulge Type** | **Potential Off-target Sequence**  ***piwil2* sgRNA2** | **Number of Mismatches** | | **Chr** | **Accession No.** | **Position** | **Str** | **Gene** |
| --- | --- | --- | --- | --- | --- | --- | --- | --- |
|  |  | Total | Bulge size |  |  |  |  |  |
| DNA | crRNA: ACGG-ATCAGTTCCTCATTGGNGG  DNA: ACGGCATCAGTTCgTCATTtGTGG | 2 | 1 | LG4 | NC_031969.2 | 31418761 | + | Intron of *noggin* (LOC100706105)* |
| RNA | crRNA: ACGGATCAGTTCCTCATTGGNGG  DNA: ACGcATC-GTTCCTCATTaGAGG | 2 | 1 | LG3 | NC_031967.2 | 32609461 | + |  |
| RNA | crRNA: ACGGATCAGTTCCTCATTGGNGG  DNA: tCGGATCAGTTCCTgA-TGGAGG | 2 | 1 | LG18 | NC_031982.2 | 882223 | - |  |
| RNA | crRNA: ACGGATCAGTTCCTCATTGGNGG  DNA: ACtGAT-AGTgCCTCATTGGTGG | 2 | 1 | LG23 | NC_031986.2 | 19247442 | - |  |

Any sites consist of 12 nt identical to the seed sequence together with PAM were considered as potential off-target, but there were no such sites in Nile tilapia genome for both sgRNA1 and 2. In addition, 1 nt bulge (DNA/RNA) together with up to 2 nt mismatches in the target sequence was listed here. Dash indicates bulge sites, lowercase is the mismatched sequence and 12 nt of seed sequence is grey-shaded. The bulge type, total numbers of mismatches, size of the bulge, the target chromosome (Chr), the position of the cleavage site, the strand (str), the gene of the off-target site are shown. *, the off-target is located in the middle of an intron sized approx. 23 kb and the exons in both sides code 5’UTR of *noggin* (LOC100706105).

**Supplementary Table S2.** The most frequent 25 mutant alleles in each phenotypic group

|  | Aligned mutant alleles | Del | Ins | Sub | Indel | Read% | Freq% | Matched alleles | |
| --- | --- | --- | --- | --- | --- | --- | --- | --- | --- |
| WT | TAAGCAAACCCTGAAGCCACGGATCAGTTCCTCATTGG*GGG*TCTGGAAAATTACTCTGGAGTACCAGCGGGT |  |  |  |  |  |  |  |  |
| A1 | TAAGCAAACCCTGAAGCCACGGATCAGTTCC----TGGGGGTCTGGAAAATTACTCTGGAGTACCAGCGGGT | 4 | 0 | 0 | **-4** | 8.0 | 100.0 | B1 | C1 |
| A2 | TAAGCAAACCCTGAAGCCACGGATCAGTTCCTCA-TGGGGGTCTGGAAAATTACTCTGGAGTACCAGCGGGT | 1 | 0 | 0 | **-1** | 7.2 | 100.0 | B2 | C2 |
| A3 | TAAGCAAACCCTGAAGCCACGGATCAGTTCCT------------GGAAAATTACTCTGGAGTACCAGCGGGT | 12 | 0 | 0 | -12 | 5.9 | 93.3 | B4 | C4 |
| A4 | TAAGCAAACCCTGAAGCCACGGATCAGTTCCTC----------TGGAAAATTACTCTGGAGTACCAGCGGGT | 10 | 0 | 0 | **-10** | 5.4 | 93.3 | B3 | C3 |
| A5 | TAAGCAAACCCTGAAGCCACGGATCAGT--------------CTGGAAAATTACTCTGGAGTACCAGCGGGT | 14 | 0 | 0 | **-14** | 6.1 | 86.7 | B8 | C5 |
| A6 | TAAGCAAACCCTGAAGCCACGGATCAGTTC-------------TGGAAAATTACTCTGGAGTACCAGCGGGT | 13 | 0 | 0 | **-13** | 3.7 | 86.7 | B5 | C11 |
| A7 | TAAGCAAACCCTGAAGCCACGGATCA-------------GGTCTGGAAAATTACTCTGGAGTACCAGCGGGT | 13 | 0 | 0 | **-13** | 2.3 | 86.7 | B6 | C10 |
| A8 | TAAGCAAACCCTGAAGCCACGGATCAG--------TGGGGGTCTGGAAAATTACTCTGGAGTACCAGCGGGT | 8 | 0 | 0 | **-8** | 1.7 | 66.7 | B18 | C21 |
| A9 | TAAGCAAACCCTGAAGCCACGGATCAGTTCCTC--TGGGGGTCTGGAAAATTACTCTGGAGTACCAGCGGGT | 2 | 0 | 0 | **-2** | 2.0 | 60.0 |  | C7 |
| A10 | TAAGCAAACCCTGAAGCCACGGATCAG-------TTGGGGGTCTGGAAAATTACTCTGGAGTACCAGCGGGT | 7 | 0 | 0 | **-7** | 1.4 | 60.0 | B14 | C8 |
| A11 | TAAGCAAACCCTGAAGCCACGGATCA----------GGGGGTCTGGAAAATTACTCTGGAGTACCAGCGGGT | 10 | 0 | 0 | **-10** | 2.5 | 40.0 | B12 | C17 |
| A12 | TAAGCAAACCCTGAAGCCACGG----------------GGGTCTGGAAAATTACTCTGGAGTACCAGCGGGT | 16 | 0 | 0 | **-16** | 1.2 | 40.0 | B10 |  |
| A13 | TAAGCAAACCCTGAAGCCACGG------------------GTCTGGAAAATTACTCTGGAGTACCAGCGGGT | 18 | 0 | 0 | -18 | 1.3 | 33.3 | B15 |  |
| A14 | TAAGCAAACCCTGAAGCCACGGATCAGTTCCTCA-------TCTGGAAAATTACTCTGGAGTACCAGCGGGT | 7 | 0 | 0 | **-7** | 1.1 | 33.3 | B22 | C14 |
| A15 | TAAGCAAACCCTGAAGCCACGGATCAGTTCCTC-----TGGTCTGGAAAATTACTCTGGAGTACCAGCGGGT | 5 | 0 | 1 | **-5** | 1.2 | 26.7 | B20 | C13 |
| A16 | TAAGCAAACCCTGAAGCCACGGATCAGTTCC-----GGGGGTCTGGAAAATTACTCTGGAGTACCAGCGGGT | 5 | 0 | 0 | **-5** | 0.6 | 26.7 |  |  |
| A17 | TAAGCAAACCCTGAAGCCACGGA------------------TCTGGAAAATTACTCTGGAGTACCAGCGGGT | 18 | 0 | 0 | -18 | 0.6 | 26.7 | B11 | C24 |
| A18 | TAAGCAAACCCTGAAGCCACGGATCA-----------GGGGTCTGGAAAATTACTCTGGAGTACCAGCGGGT | 11 | 0 | 0 | **-11** | 0.5 | 26.7 |  | C22 |
| A19 | TAAGCAAACCCTGAAGCCACGG-------------------TCTGGAAAATTACTCTGGAGTACCAGCGGGT | 19 | 0 | 0 | **-19** | 0.4 | 26.7 | B21 |  |
| A20 | TAAGCAAACCCTGAAGCCACGGATCAGTTCCT-------GGTCTGGAAAATTACTCTGGAGTACCAGCGGGT | 7 | 0 | 0 | **-7** | 0.6 | 20.0 |  | C15 |
| A21 | TAAGCAAACCCTGAAGCCACGGATCAGTTC-----TGGGGGTCTGGAAAATTACTCTGGAGTACCAGCGGGT | 5 | 0 | 0 | **-5** | 0.4 | 20.0 | B24 |  |
| A22 | TAAGCAAACCCTGAAGCCACGG-----------------GGTCTGGAAAATTACTCTGGAGTACCAGCGGGT | 17 | 0 | 0 | **-17** | 0.4 | 20.0 | B23 |  |
| A23 | TAAGCAAACCCTGAAGCCACGGATCAGTTCCTCA------GTCTGGAAAATTACTCTGGAGTACCAGCGGGT | 6 | 0 | 0 | -6 | 0.3 | 20.0 |  | C23 |
| A24 | TAAGCAAACCCTG------------------------GGGGTCTGGAAAATTACTCTGGAGTACCAGCGGGT | 24 | 0 | 0 | **-24** | 0.2 | 20.0 |  |  |
| A25 | TAAGCAAACCCTGAAGCCACGGATCAGTTCCTCA-----GGTCTGGAAAATTACTCTGGAGTACCAGCGGGT | 5 | 0 | 0 | **-5** | 0.2 | 20.0 | B13 | C6 |
| B1 | TAAGCAAACCCTGAAGCCACGGATCAGTTCC----TGGGGGTCTGGAAAATTACTCTGGAGTACCAGCGGGT | 4 | 0 | 0 | **-4** | 7.2 | 92.3 | A1 | C1 |
| B2 | TAAGCAAACCCTGAAGCCACGGATCAGTTCCTCA-TGGGGGTCTGGAAAATTACTCTGGAGTACCAGCGGGT | 1 | 0 | 0 | **-1** | 6.1 | 92.3 | A2 | C2 |
| B3 | TAAGCAAACCCTGAAGCCACGGATCAGTTCCTC----------TGGAAAATTACTCTGGAGTACCAGCGGGT | 10 | 0 | 0 | **-10** | 5.6 | 76.9 | A4 | C3 |
| B4 | TAAGCAAACCCTGAAGCCACGGATCAGTTCCT------------GGAAAATTACTCTGGAGTACCAGCGGGT | 12 | 0 | 0 | -12 | 3.9 | 69.2 | A3 | C4 |
| B5 | TAAGCAAACCCTGAAGCCACGGATCAGTTC-------------TGGAAAATTACTCTGGAGTACCAGCGGGT | 13 | 0 | 0 | **-13** | 2.2 | 61.5 | A6 | C11 |
| B6 | TAAGCAAACCCTGAAGCCACGGATCA-------------GGTCTGGAAAATTACTCTGGAGTACCAGCGGGT | 13 | 0 | 0 | **-13** | 3.2 | 53.8 | A7 | C10 |
| B7 | TAAGCAAACCCTGAAGCCACGGATCAGTTCCTCAGTTCCTGGGGGTCTGGAAAATTACTCTGGAGTACCAGCGGGT | 0 | 4 | 1 | **4** | 4.0 | 46.2 |  | C12 |
| B8 | TAAGCAAACCCTGAAGCCACGGATCAGT--------------CTGGAAAATTACTCTGGAGTACCAGCGGGT | 14 | 0 | 0 | **-14** | 2.0 | 46.2 | A5 | C5 |
| B9 | TAAGCAAACCCTGAAGCCACGGATCAGTTCCTC-CTGGGGGTCTGGAAAATTACTCTGGAGTACCAGCGGGT | 1 | 0 | 1 | **-1** | 1.0 | 38.5 |  | C9 |
| B10 | TAAGCAAACCCTGAAGCCACGG----------------GGGTCTGGAAAATTACTCTGGAGTACCAGCGGGT | 16 | 0 | 0 | **-16** | 0.7 | 38.5 | A12 |  |
| B11 | TAAGCAAACCCTGAAGCCACGGA------------------TCTGGAAAATTACTCTGGAGTACCAGCGGGT | 18 | 0 | 0 | -18 | 2.8 | 30.8 | A17 | C24 |
| B12 | TAAGCAAACCCTGAAGCCACGGATCA----------GGGGGTCTGGAAAATTACTCTGGAGTACCAGCGGGT | 10 | 0 | 0 | **-10** | 1.1 | 30.8 | A11 | C17 |
| B13 | TAAGCAAACCCTGAAGCCACGGATCAGTTCCTCA-----GGTCTGGAAAATTACTCTGGAGTACCAGCGGGT | 5 | 0 | 0 | **-5** | 0.9 | 30.8 | A25 | C6 |
| B14 | TAAGCAAACCCTGAAGCCACGGATCAG-------TTGGGGGTCTGGAAAATTACTCTGGAGTACCAGCGGGT | 7 | 0 | 0 | **-7** | 0.5 | 30.8 |  |  |
| B15 | TAAGCAAACCCTGAAGCCACGG------------------GTCTGGAAAATTACTCTGGAGTACCAGCGGGT | 18 | 0 | 0 | -18 | 0.3 | 30.8 | A13 |  |
| B16 | TAAGCAAACCCTGAAGCCACGGATCAGTTCCTCCGTGGGGGTCTGGAAAATTACTCTGGAGTACCAGCGGGT | 0 | 0 | 2 | 0 | 1.0 | 23.1 |  |  |
| B17 | TAAGCAAACCCTGAAGCCACGGATCAGTTCCTCA---------TGGAAAATTACTCTGGAGTACCAGCGGGT | 9 | 0 | 0 | -9 | 0.9 | 23.1 |  |  |
| B18 | TAAGCAAACCCTGAAGCCACGGATCAG--------TGGGGGTCTGGAAAATTACTCTGGAGTACCAGCGGGT | 8 | 0 | 0 | **-8** | 0.7 | 23.1 | A8 | C21 |
| B19 | TAAGCAAACCCTGAAGCCACGGATCAGTTCCT-----GGGGTCTGGAAAATTACTCTGGAGTACCAGCGGGT | 5 | 0 | 0 | **-5** | 0.6 | 23.1 |  | C18 |
| B20 | TAAGCAAACCCTGAAGCCACGGATCAGTTCCTC-----TGGTCTGGAAAATTACTCTGGAGTACCAGCGGGT | 5 | 0 | 1 | **-5** | 0.4 | 23.1 | A15 | C13 |
| B21 | TAAGCAAACCCTGAAGCCACGG-------------------TCTGGAAAATTACTCTGGAGTACCAGCGGGT | 19 | 0 | 0 | **-19** | 0.3 | 23.1 | A19 |  |
| B22 | TAAGCAAACCCTGAAGCCACGGATCAGTTCCTCA-------TCTGGAAAATTACTCTGGAGTACCAGCGGGT | 7 | 0 | 0 | **-7** | 1.3 | 15.4 | A14 | C14 |
| B23 | TAAGCAAACCCTGAAGCCACGG-----------------GGTCTGGAAAATTACTCTGGAGTACCAGCGGGT | 17 | 0 | 0 | **-17** | 0.8 | 15.4 | A22 |  |
| B24 | TAAGCAAACCCTGAAGCCACGGATCAGTTC-----TGGGGGTCTGGAAAATTACTCTGGAGTACCAGCGGGT | 5 | 0 | 0 | **-5** | 0.7 | 15.4 | A21 |  |
| B25 | TAAGCAAACCCTGAAGCCACGGATCAGTTCCAC------GGTCTGGAAAATTACTCTGGAGTACCAGCGGGT | 6 | 0 | 1 | -6 | 0.5 | 15.4 |  |  |
| C1 | TAAGCAAACCCTGAAGCCACGGATCAGTTCC----TGGGGGTCTGGAAAATTACTCTGGAGTACCAGCGGGT | 4 | 0 | 0 | **-4** | 8.4 | 100.0 | A1 | B1 |
| C2 | TAAGCAAACCCTGAAGCCACGGATCAGTTCCTCA-TGGGGGTCTGGAAAATTACTCTGGAGTACCAGCGGGT | 1 | 0 | 0 | **-1** | 5.8 | 95.8 | A2 | B2 |
| C3 | TAAGCAAACCCTGAAGCCACGGATCAGTTCCTC----------TGGAAAATTACTCTGGAGTACCAGCGGGT | 10 | 0 | 0 | **-10** | 6.6 | 91.7 | A4 | B3 |
| C4 | TAAGCAAACCCTGAAGCCACGGATCAGTTCCT------------GGAAAATTACTCTGGAGTACCAGCGGGT | 12 | 0 | 0 | -12 | 3.3 | 70.8 | A3 | B4 |
| C5 | TAAGCAAACCCTGAAGCCACGGATCAGT--------------CTGGAAAATTACTCTGGAGTACCAGCGGGT | 14 | 0 | 0 | **-14** | 1.3 | 50.0 | A5 | B8 |
| C6 | TAAGCAAACCCTGAAGCCACGGATCAGTTCCTCA-----GGTCTGGAAAATTACTCTGGAGTACCAGCGGGT | 5 | 0 | 0 | **-5** | 1.3 | 41.7 | A25 | B13 |
| C7 | TAAGCAAACCCTGAAGCCACGGATCAGTTCCTC--TGGGGGTCTGGAAAATTACTCTGGAGTACCAGCGGGT | 2 | 0 | 0 | **-2** | 1.2 | 37.5 | A9 |  |
| C8 | TAAGCAAACCCTGAAGCCACGGATCAG-------TTGGGGGTCTGGAAAATTACTCTGGAGTACCAGCGGGT | 7 | 0 | 0 | **-7** | 0.9 | 37.5 | A10 | B14 |
| C9 | TAAGCAAACCCTGAAGCCACGGATCAGTTCCTC-CTGGGGGTCTGGAAAATTACTCTGGAGTACCAGCGGGT | 1 | 0 | 1 | **-1** | 1.8 | 33.3 |  | B9 |
| C10 | TAAGCAAACCCTGAAGCCACGGATCA-------------GGTCTGGAAAATTACTCTGGAGTACCAGCGGGT | 13 | 0 | 0 | **-13** | 1.7 | 33.3 | A7 | B6 |
| C11 | TAAGCAAACCCTGAAGCCACGGATCAGTTC-------------TGGAAAATTACTCTGGAGTACCAGCGGGT | 13 | 0 | 0 | **-13** | 1.6 | 29.2 | A6 | B5 |
| C12 | TAAGCAAACCCTGAAGCCACGGATCAGTTCCTCAGTTCCTGGGGGTCTGGAAAATTACTCTGGAGTACCAGCGGGT | 0 | 4 | 1 | **4** | 0.9 | 29.2 |  | B7 |
| C13 | TAAGCAAACCCTGAAGCCACGGATCAGTTCCTC-----TGGTCTGGAAAATTACTCTGGAGTACCAGCGGGT | 5 | 0 | 1 | **-5** | 1.3 | 25.0 | A15 | B20 |
| C14 | TAAGCAAACCCTGAAGCCACGGATCAGTTCCTCA-------TCTGGAAAATTACTCTGGAGTACCAGCGGGT | 7 | 0 | 0 | **-7** | 1.3 | 20.8 | A14 | B22 |
| C15 | TAAGCAAACCCTGAAGCCACGGATCAGTTCCT-------GGTCTGGAAAATTACTCTGGAGTACCAGCGGGT | 7 | 0 | 0 | **-7** | 1.0 | 20.8 | A20 |  |
| C16 | TAAGCAAACCCTGAAGCCAC----------------GGGGGTCTGGAAAATTACTCTGGAGTACCAGCGGGT | 16 | 0 | 0 | **-16** | 0.8 | 20.8 |  |  |
| C17 | TAAGCAAACCCTGAAGCCACGGATCA----------GGGGGTCTGGAAAATTACTCTGGAGTACCAGCGGGT | 10 | 0 | 0 | **-10** | 0.8 | 20.8 | A11 | B12 |
| C18 | TAAGCAAACCCTGAAGCCACGGATCAGTTCCT-----GGGGTCTGGAAAATTACTCTGGAGTACCAGCGGGT | 5 | 0 | 0 | **-5** | 0.4 | 20.8 |  | B19 |
| C19 | TAAGCAAACCCTGAAGCCACGGATCAGTTCCTCATTTGGGGGTCTGGAAAATTACTCTGGAGTACCAGCGGGT | 0 | 1 | 0 | **1** | 1.3 | 16.7 |  |  |
| C20 | TAAGCAAACCCTGAAGCCACGGATCAGTTCCTCA----GGGTCTGGAAAATTACTCTGGAGTACCAGCGGGT | 4 | 0 | 0 | **-4** | 1.0 | 16.7 |  |  |
| C21 | TAAGCAAACCCTGAAGCCACGGATCAG--------TGGGGGTCTGGAAAATTACTCTGGAGTACCAGCGGGT | 8 | 0 | 0 | **-8** | 0.9 | 16.7 | A8 | B18 |
| C22 | TAAGCAAACCCTGAAGCCACGGATCA-----------GGGGTCTGGAAAATTACTCTGGAGTACCAGCGGGT | 11 | 0 | 0 | **-11** | 0.8 | 16.7 | A18 |  |
| C23 | TAAGCAAACCCTGAAGCCACGGATCAGTTCCTCA------GTCTGGAAAATTACTCTGGAGTACCAGCGGGT | 6 | 0 | 0 | -6 | 0.6 | 16.7 | A23 |  |
| C24 | TAAGCAAACCCTGAAGCCACGGA------------------TCTGGAAAATTACTCTGGAGTACCAGCGGGT | 18 | 0 | 0 | -18 | 0.4 | 16.7 | A17 | B11 |
| C25 | TAAGCAAACCCTGAAGCCACGGATCAGTTCCTCATCTGGGGTCTGGAAAATTACTCTGGAGTACCAGCGGGT | 0 | 0 | 2 | 0 | 1.9 | 12.5 |  |  |

Del, deletion (bp); Ins, Insertion (bp); Sub, substitution (bp), Indel, size of indel (bp); Read%, average reads per fish (%); Freq%, frequency in each phenotypic group (%); WT, wildtype. Grey shaded = guide sequence; italic = PAM; dash = deletion; blue coloured = predicted microhomology; red coloured = substitution; boxed = insertion; bold indel value = frame-shifted indel.

**Supplementary Table S3.** Percentiles (5^th^, 25^th^, 50^th^, 75^th^ and 95^th^) of indel size distribution in each phenotype group

|  | **5th** | **25th** | **50th** | **75^th^** | **95th** |
| --- | --- | --- | --- | --- | --- |
| Phenotype A | -25 | -14 | -7 | -1 | 8.4 |
| Phenotype B | -23 | -12 | -5 | 0 | 11.2 |
| Phenotype C | -32.4 | -12 | -5 | 1 | 12 |

**Supplementary Table S4**. List of mutants which were outliers in regression analysis between fragment analysis and NGS shown in Fig. 4A. WT length mutated sequences are mutant sequences without changes in absolute length. del, deletion (bp); ins, insertion (bp); mut, mutation (bp); sub, substitution

|  | WT length (%) in fragment analysis | WT (%) in NGS | WT length mutant seq (%) in NGS (No. of seq) | Description of WT length mutated seq | WT + WT length mutants (%) in NGS |
| --- | --- | --- | --- | --- | --- |
| mutant 28 | 34.1 | 0.0 | 36.6 (2) | 33.3% of 2 del, 2 ins and 2 mut 3.3% of 1 sub | 36.6 |
| mutant 42 | 33.9 | 18.1 | 12.0 (3) | 7.9% of 2 mut  3.1% of 2 mut  1% of 3 del, 3 ins and 3 mut | 30.1 |
| mutant 51 | 51.6 | 1.3 | 51.8 (2) | 42.4% of 2 mut  9.4% of 3 del, 3 ins and 2 mut | 53.0 |
| mutant 52 | 27.6 | 0.0 | 31.0 | 29.1% of 3 mut  1.9% of 1 del, 1 ins | 31.0 |

**
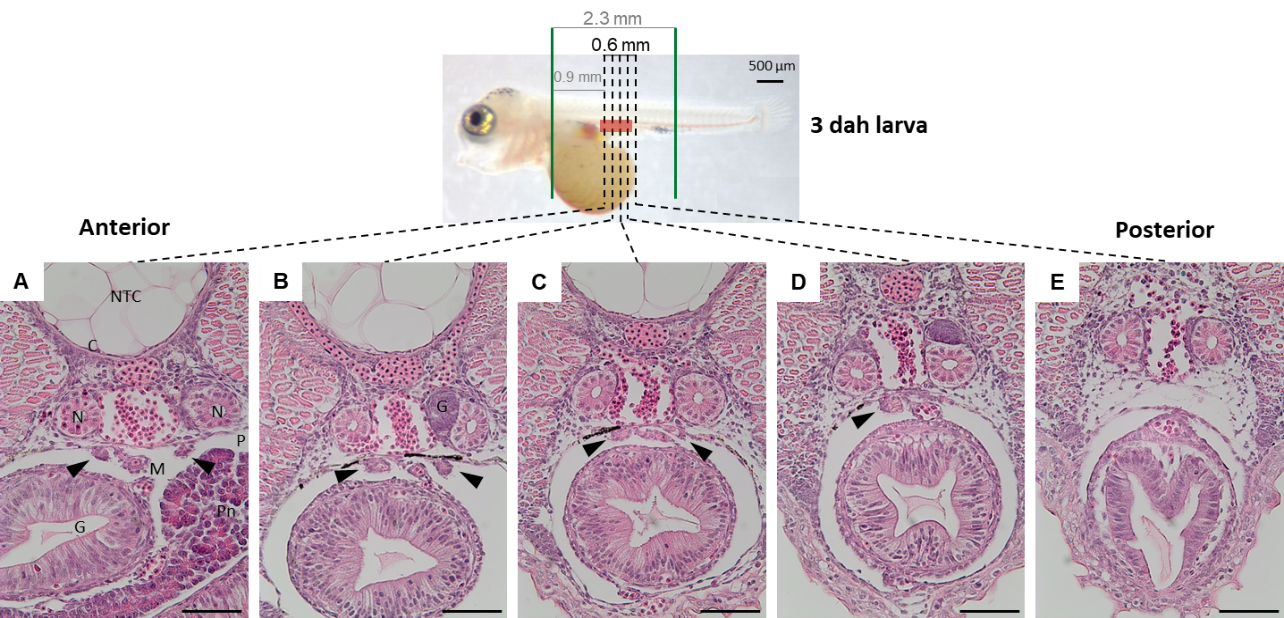
**

**Supplementary Figure 1**

(A) Deletion all 52 mutants (B) Deletion phenotype A, B &C


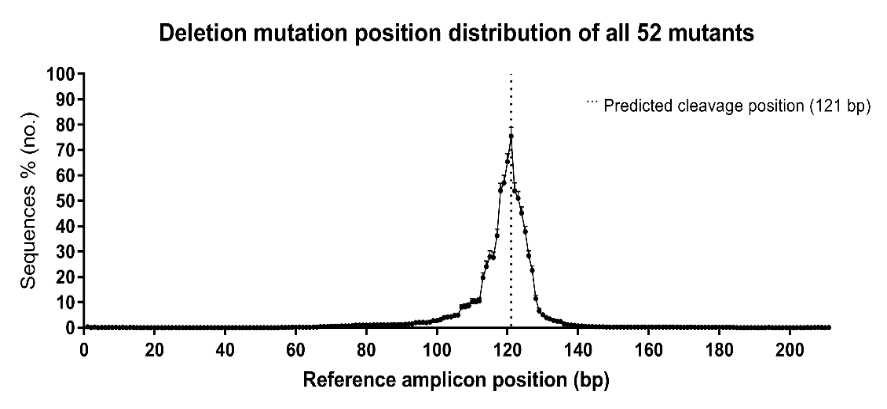

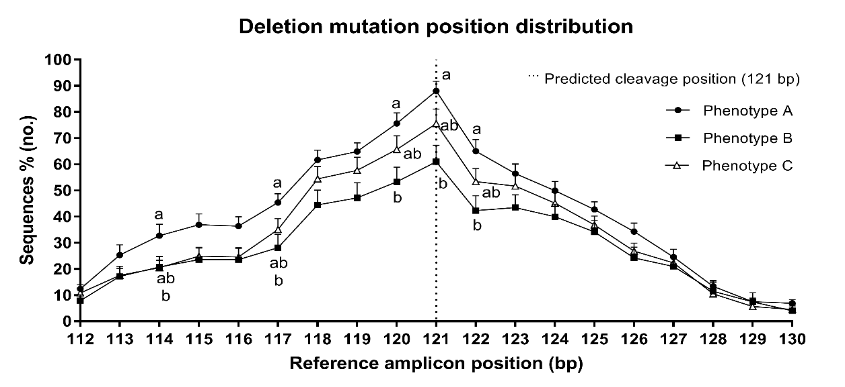


(C) Insertion al 52 mutants **(**D) Insertion phenotype A, B & C


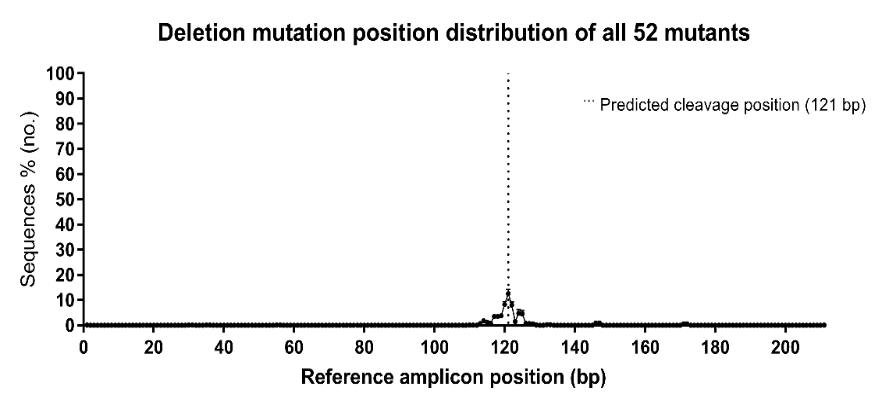

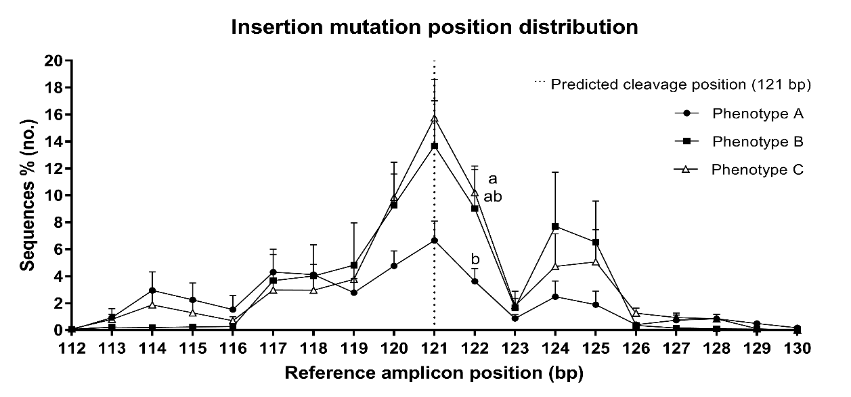


(E) Substitution al 52 mutants (F) Substitution phenotype A, B & C

**
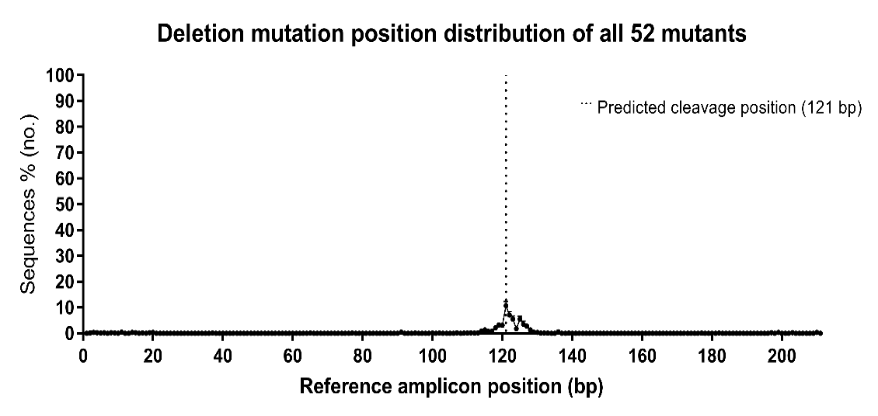
** **
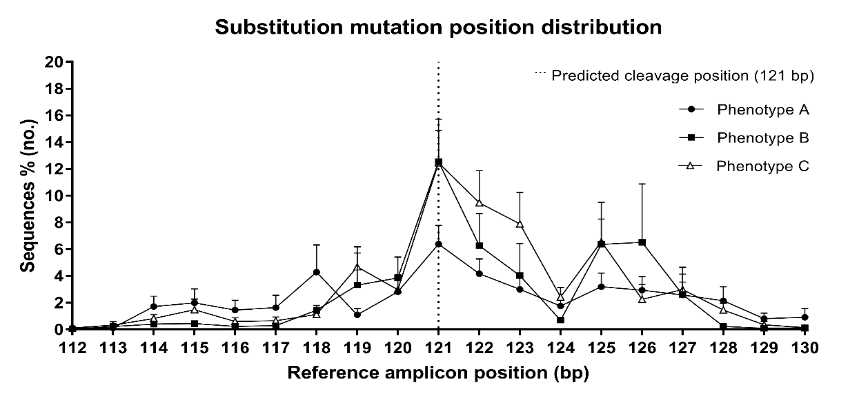
**

**Supplementary Figure S2**

(A) Frequency of indel size


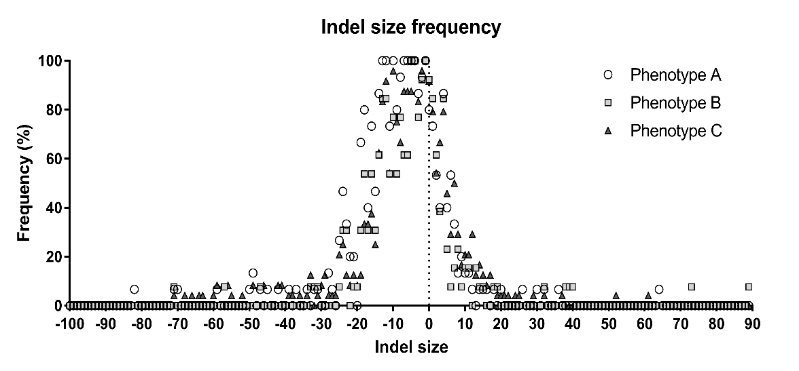


(B) Indel size (5^th^ and 95^th^ percentiles)


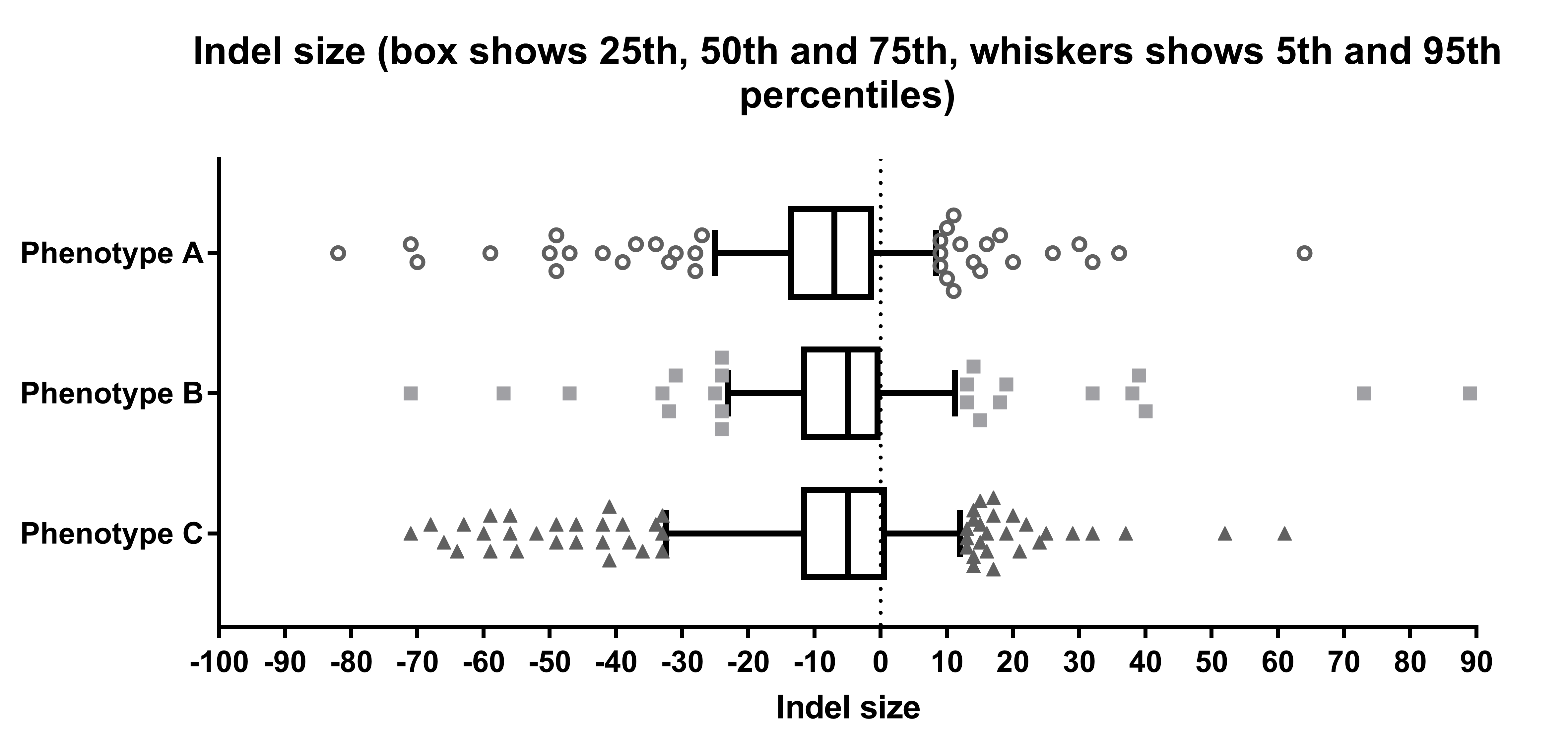


**Supplementary Figure S3**


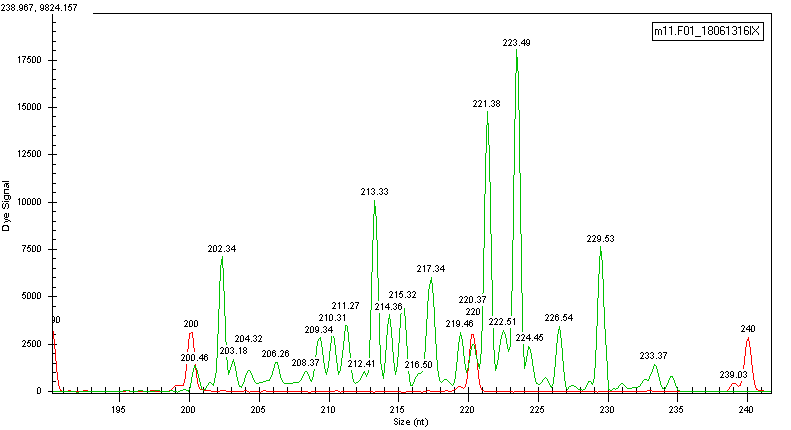

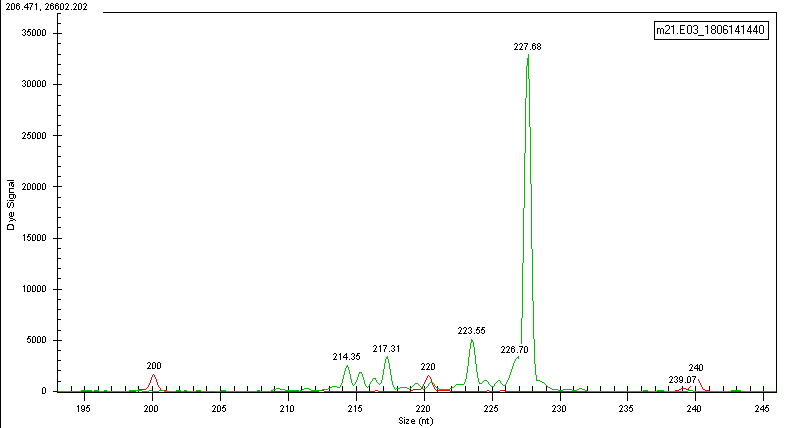

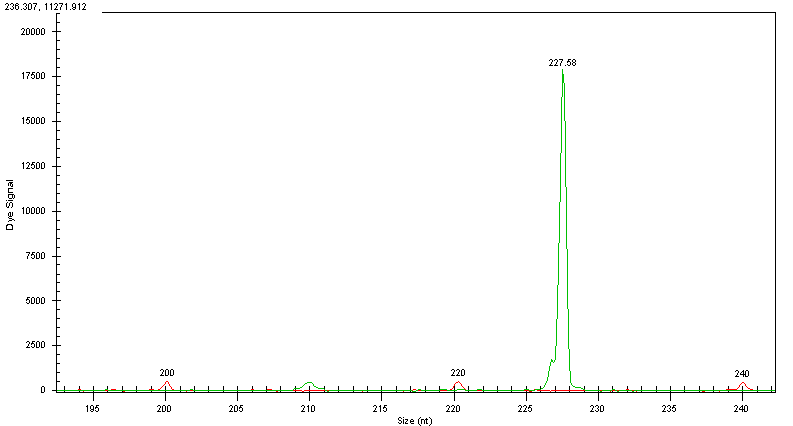


(A) WT (control 7)

(B) Mutant without WT length fragment (mutant 11, phenotype A)

(C) Mutant with WT length fragment (mutant 21, phenotype B)

*

deletion

insertion

deletion

*

**Supplementary Figure S4**


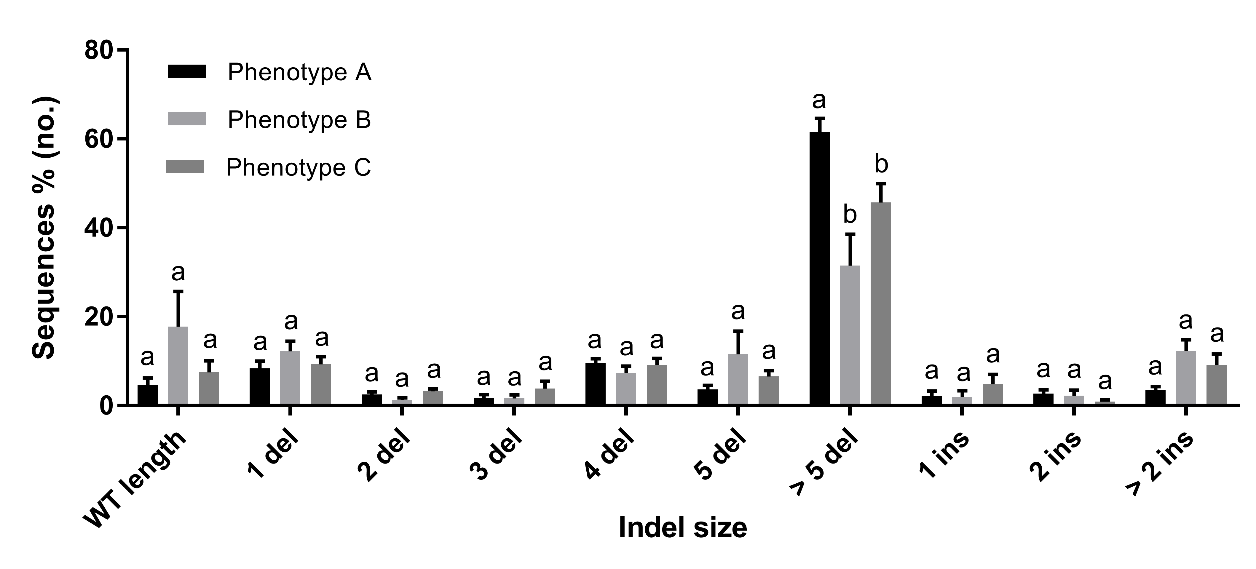


**Supplementary Figure S5**

(A) Mutant without WT length fragment (mutant 11, Phenotype A) (B) Mutant with WT length fragment (mutant 21, Phenotype B)


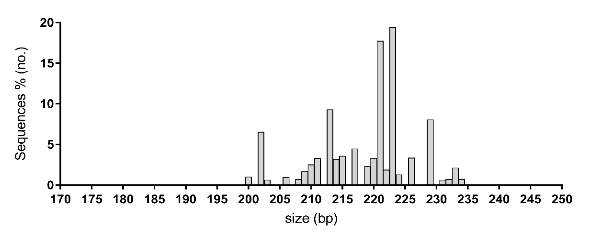

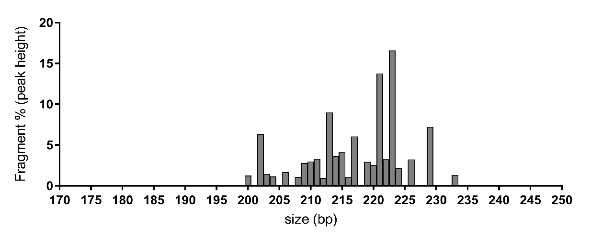


NGS

Fragment analysis

deletion

insertion

deletion

insertion


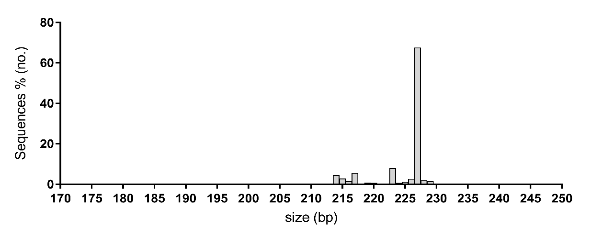

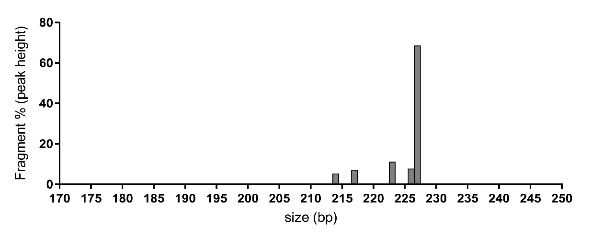


NGS

Fragment analysis

*

*

deletion

insertion

deletion

**Supplementary Figure S6**

(A) Melt curves of the 52 mutants and controls

**
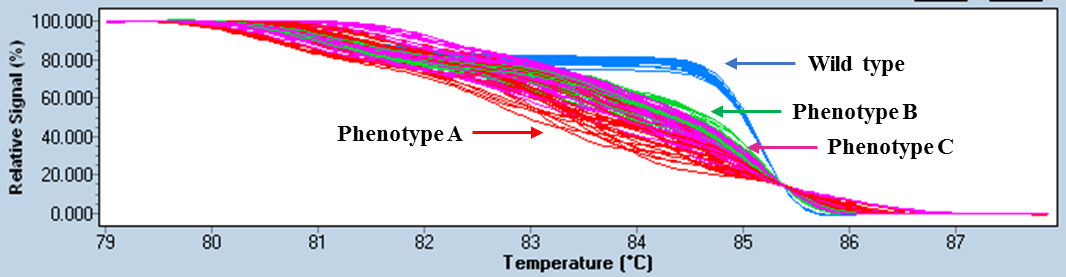
**


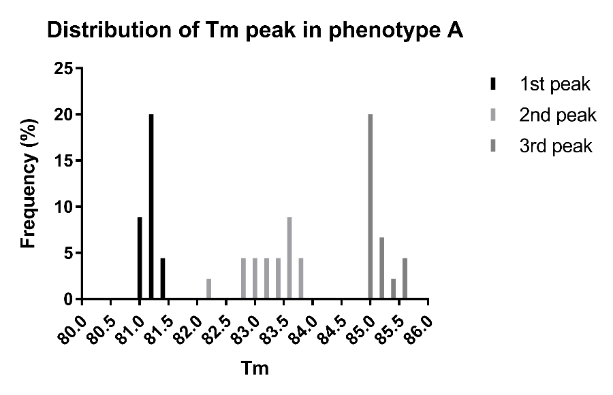

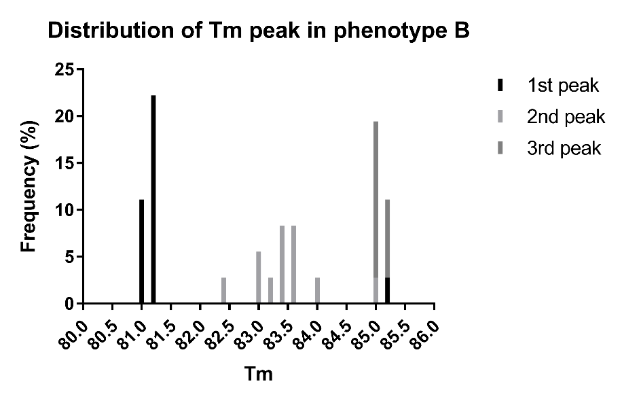

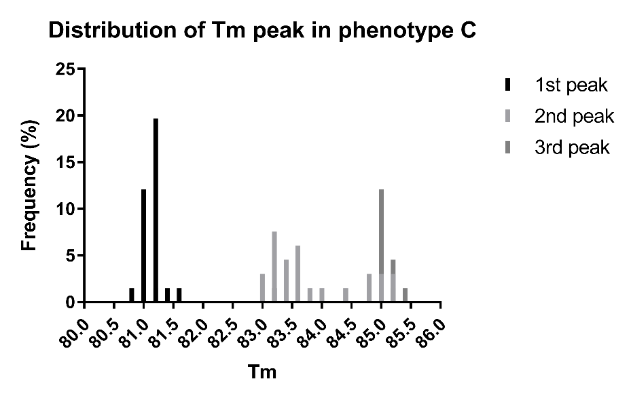


(B) WT control

(D) Phenotype B

(E) Phenotype C


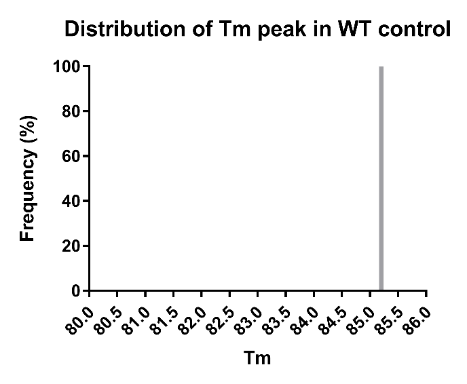


(C) Phenotype A


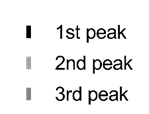

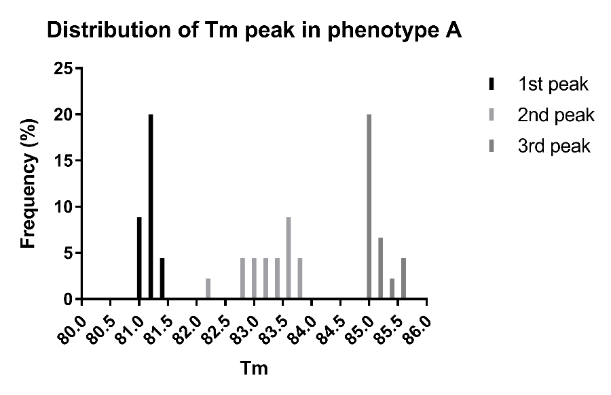

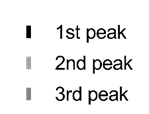


**Supplementary Figure S7**
