# Supplementary figures and images for: Physiological impact and comparison of mutant screening methods in piwil2 KO founder Nile tilapia produced by CRISPR/Cas9 system
Source: Sci Rep. 2020 Jul 28;10:12600. doi: 10.1038/s41598-020-69421-0 (PMC7387559; doi:10.1038/s41598-020-69421-0)

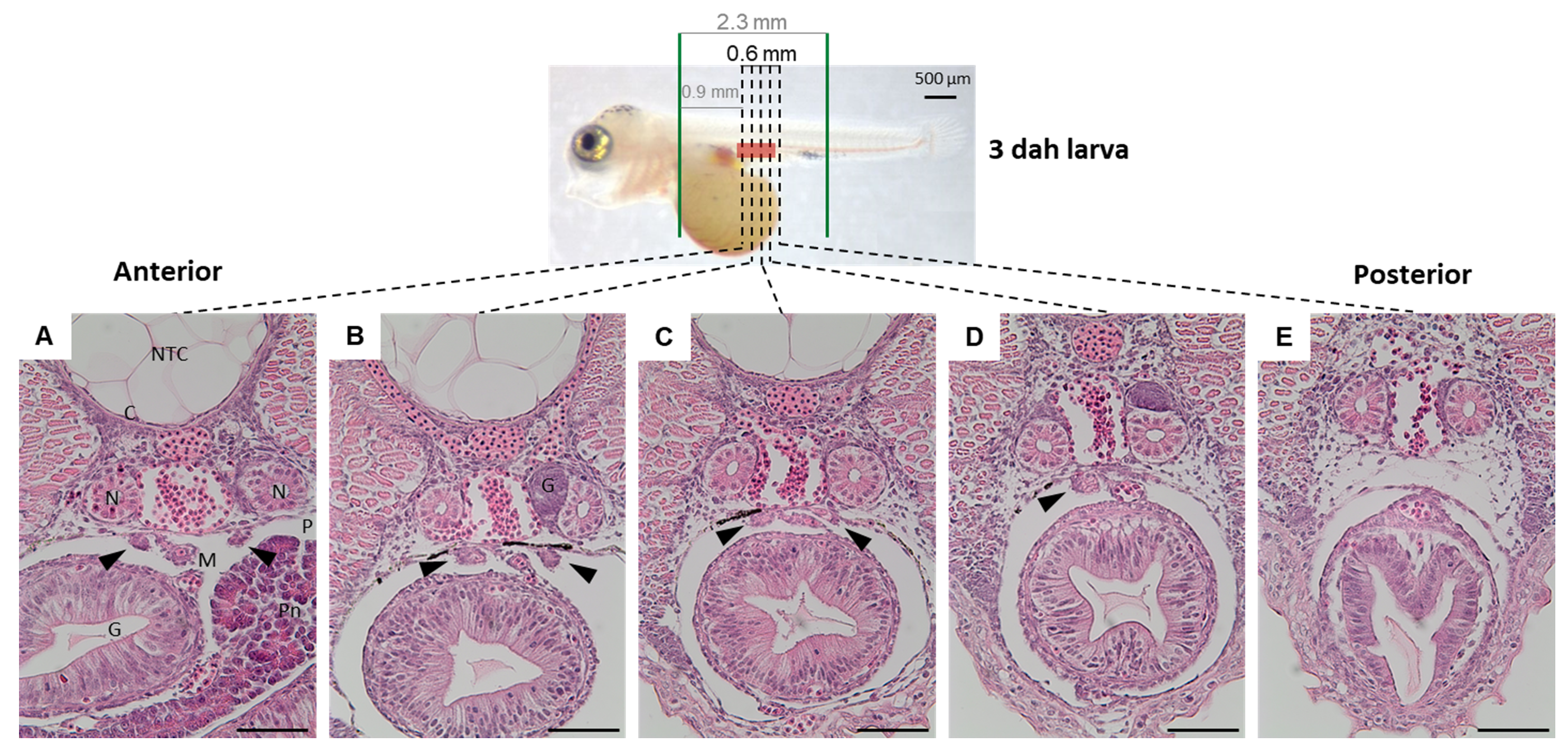

Supplement: Supplementary file 2 — Supplementary Figure S1. [file 41598_2020_69421_MOESM2_ESM.tif]

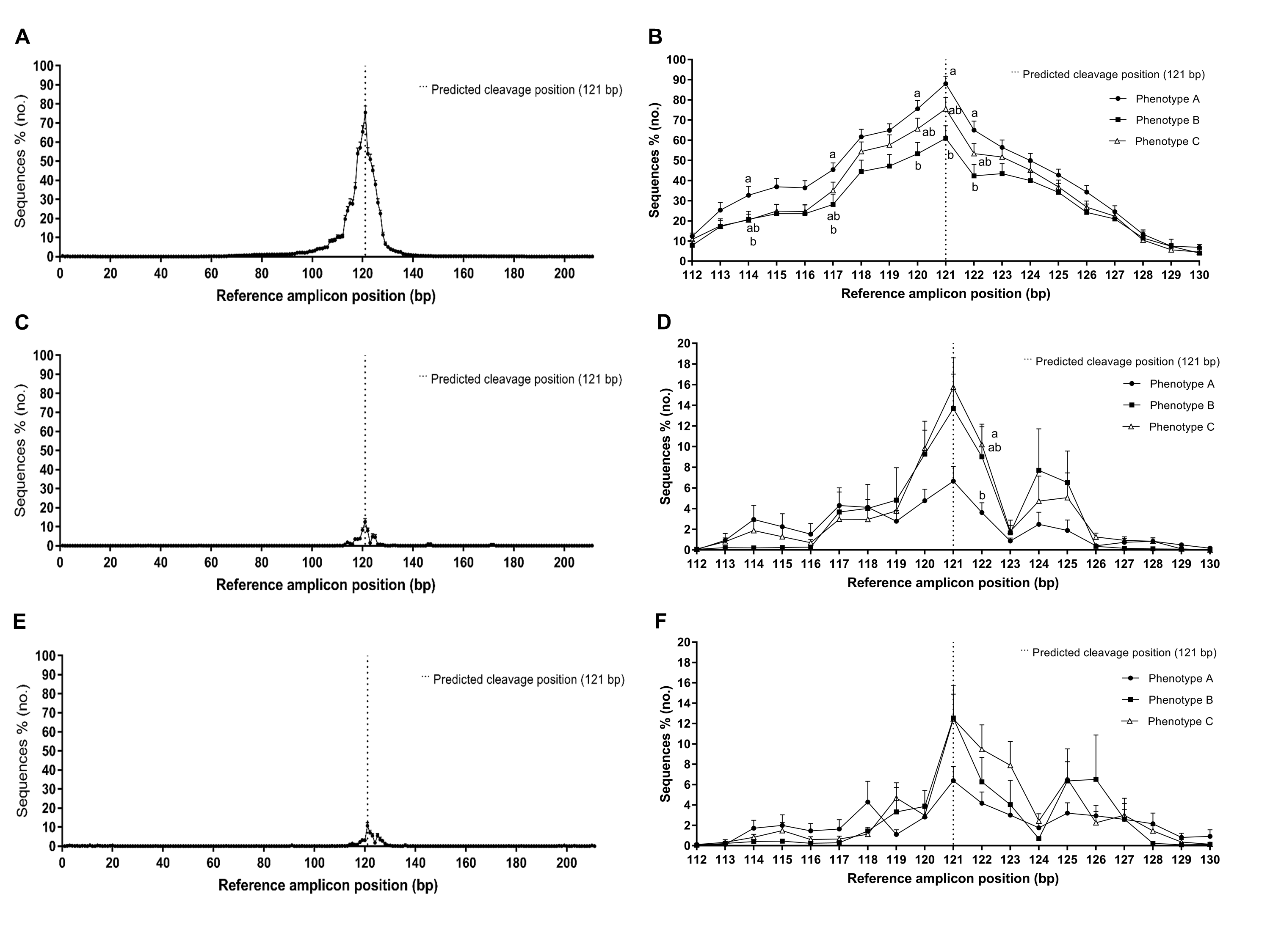

Supplement: Supplementary file 3 — Supplementary Figure S2. [file 41598_2020_69421_MOESM3_ESM.tif]

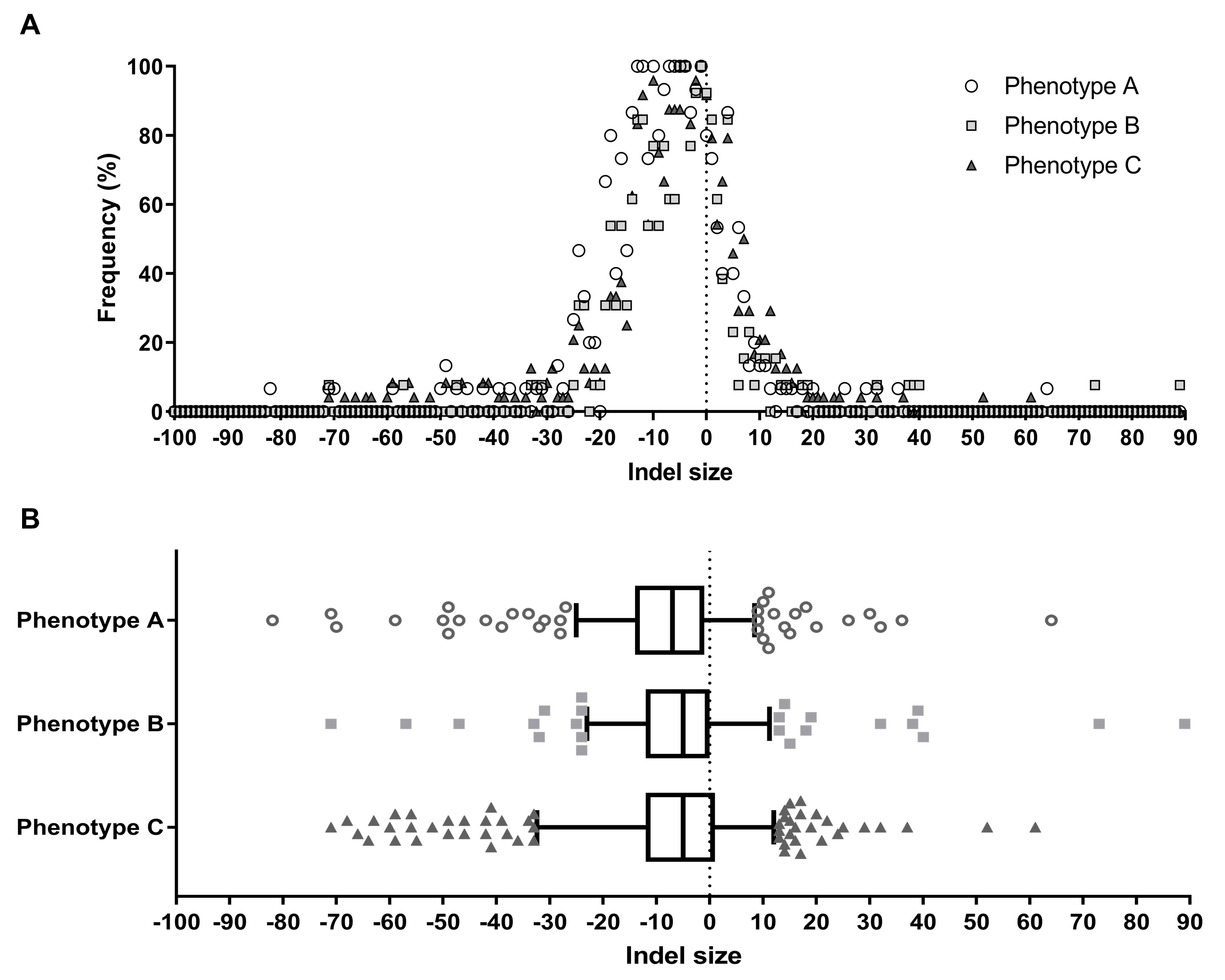

Supplement: Supplementary file 4 — Supplementary Figure S3. [file 41598_2020_69421_MOESM4_ESM.tif]

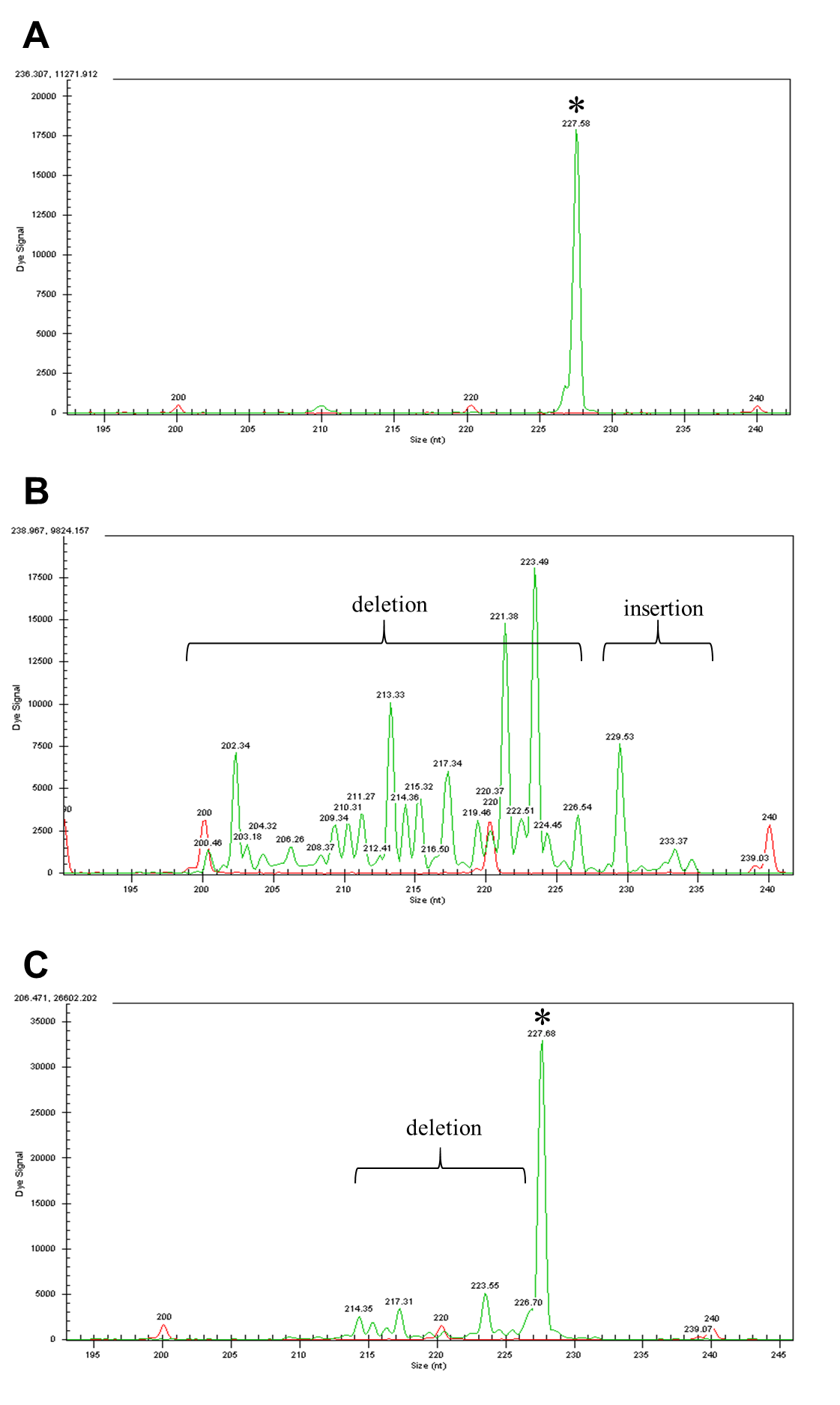

Supplement: Supplementary file 5 — Supplementary Figure S4. [file 41598_2020_69421_MOESM5_ESM.tif]

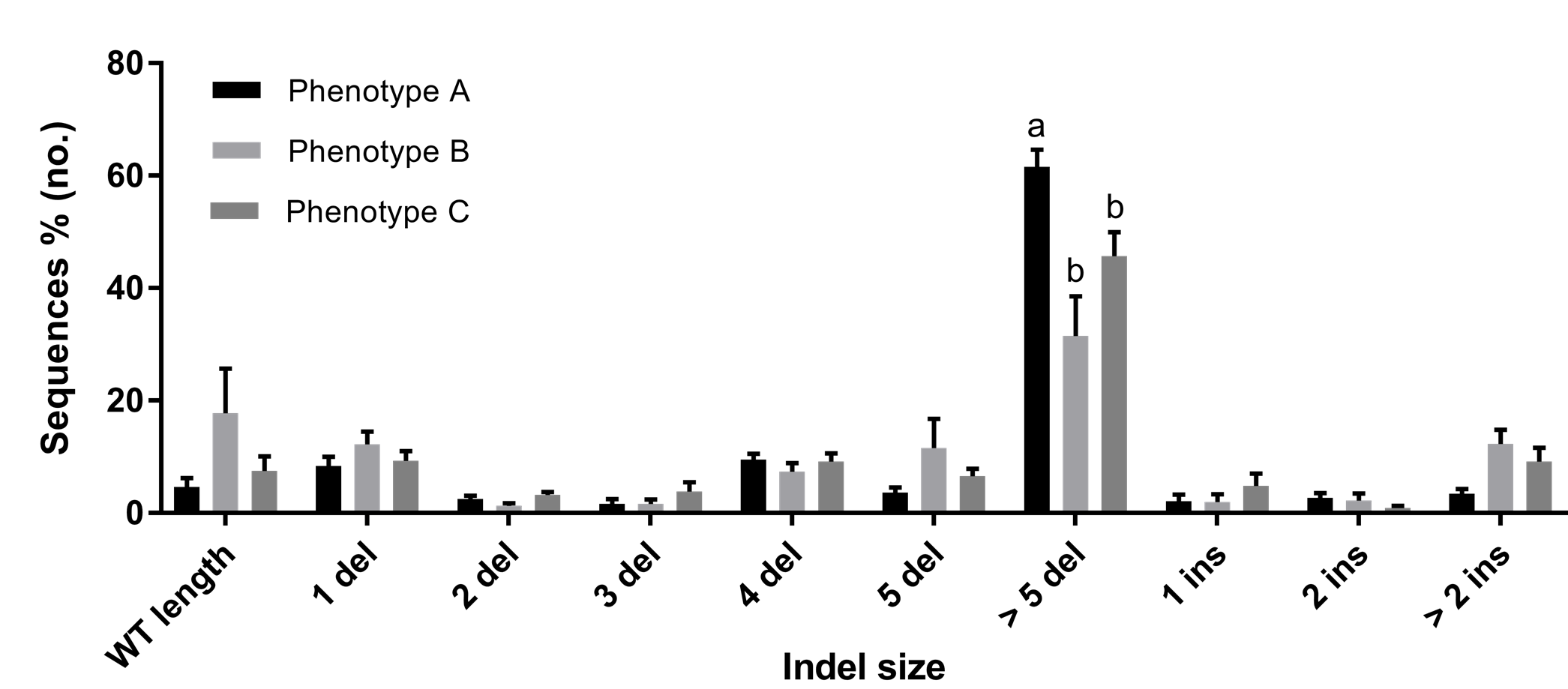

Supplement: Supplementary file 6 — Supplementary Figure S5. [file 41598_2020_69421_MOESM6_ESM.tif]

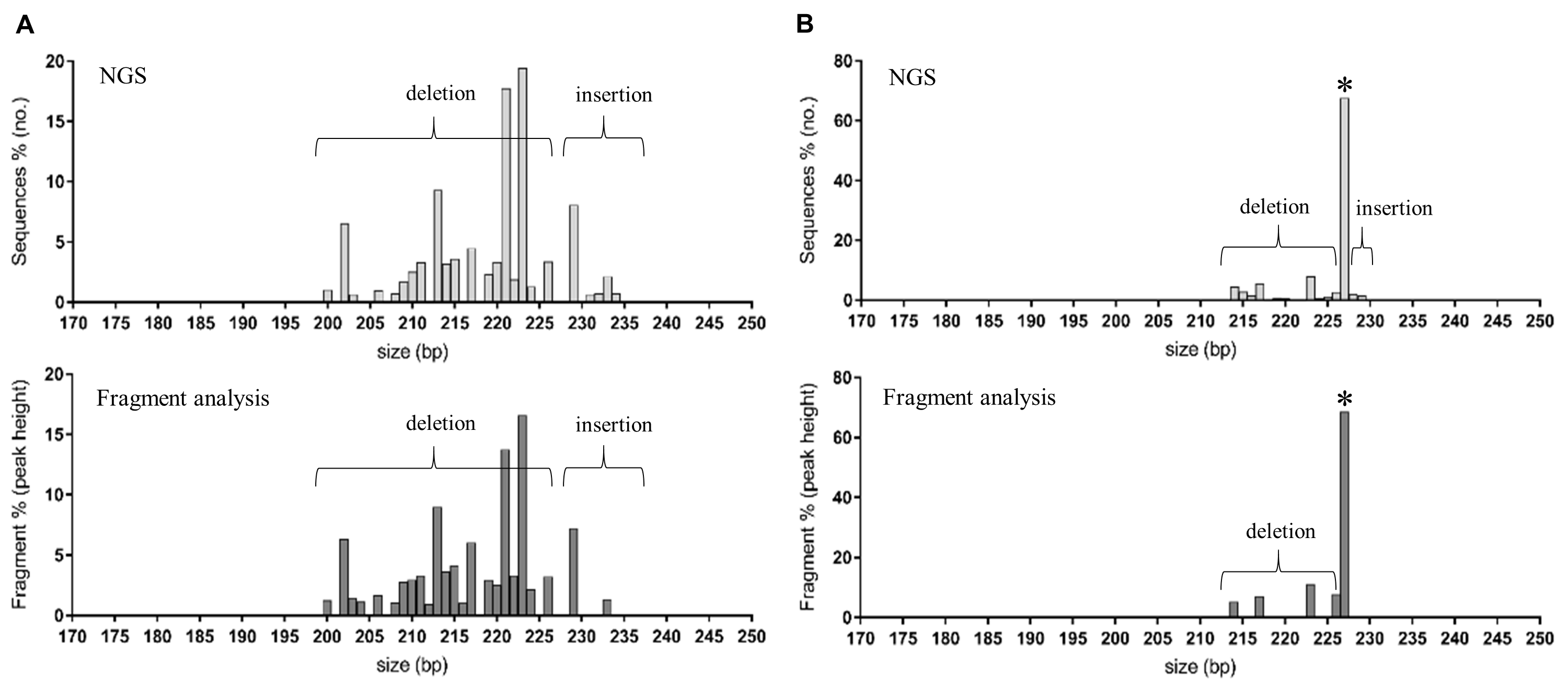

Supplement: Supplementary file 7 — Supplementary Figure S6. [file 41598_2020_69421_MOESM7_ESM.tif]

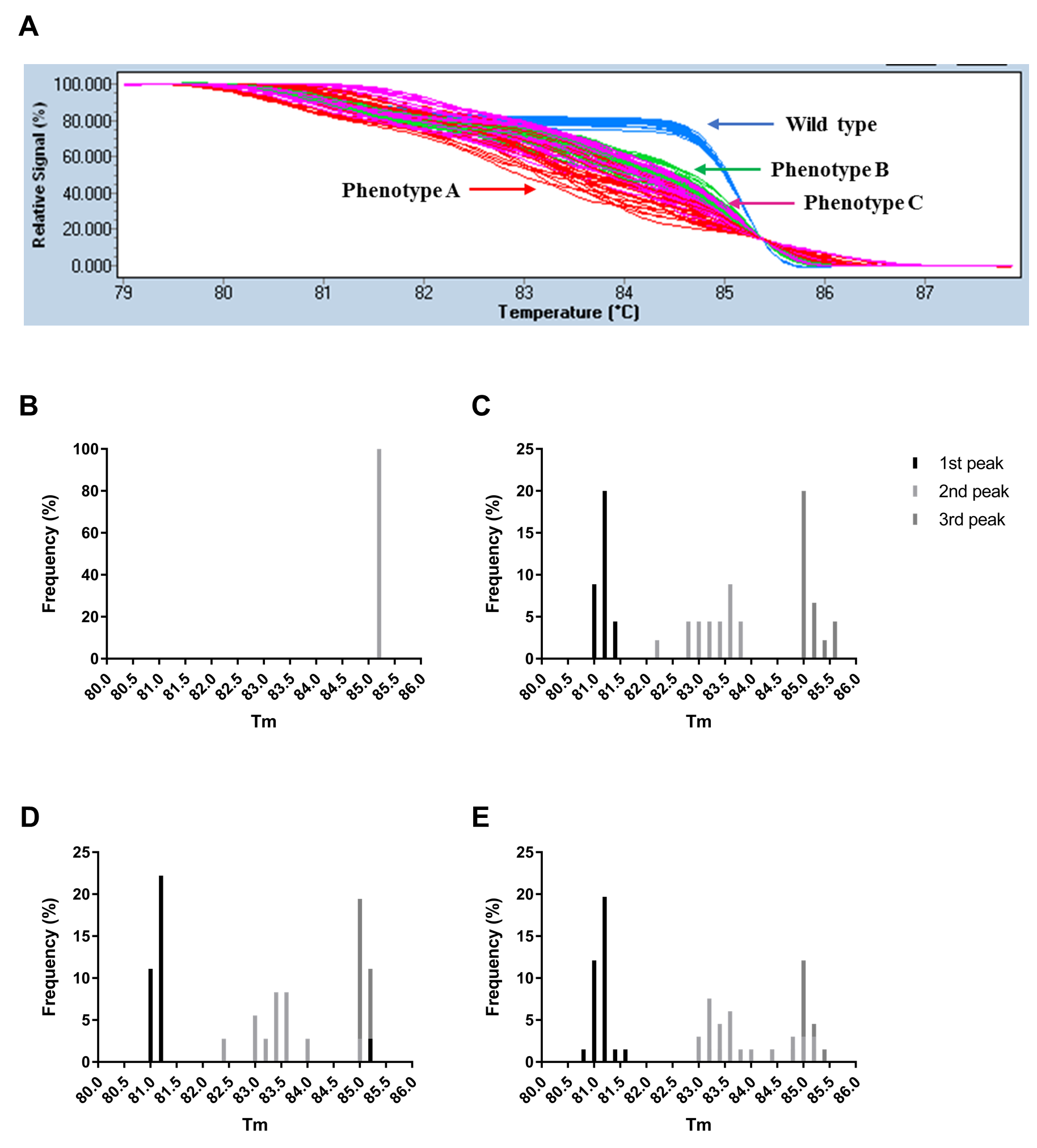

Supplement: Supplementary file 8 — Supplementary Figure S7. [file 41598_2020_69421_MOESM8_ESM.tif]
